# Multi-Omics Analysis of the Effects of Smoking on Human Tumors

**DOI:** 10.3389/fmolb.2021.704910

**Published:** 2021-11-02

**Authors:** Rui Wang, Shanshan Li, Wen Wen, Jianquan Zhang

**Affiliations:** ^1^ Department of Hepatobiliary Surgery, Affiliated Haikou Hospital of Xiangya Medical College, Central South University, Haikou, China; ^2^ Department of Nursing, Affiliated Haikou Hospital of Xiangya Medical College, Central South University, Haikou, China

**Keywords:** tobacco, cessation, former smokers, current smokers, TCGA, bioinformatics, multi-omics

## Abstract

Comprehensive studies on cancer patients with different smoking histories, including non-smokers, former smokers, and current smokers, remain elusive. Therefore, we conducted a multi-omics analysis to explore the effect of smoking history on cancer patients. Patients with smoking history were screened from The Cancer Genome Atlas database, and their multi-omics data and clinical information were downloaded. A total of 2,317 patients were included in this study, whereby current smokers presented the worst prognosis, followed by former smokers, while non-smokers showed the best prognosis. More importantly, smoking history was an independent prognosis factor. Patients with different smoking histories exhibited different immune content, and former smokers had the highest immune cells and tumor immune microenvironment. Smokers are under a higher incidence of genomic instability that can be reversed following smoking cessation in some changes. We also noted that smoking reduced the sensitivity of patients to chemotherapeutic drugs, whereas smoking cessation can reverse the situation. Competing endogenous RNA network revealed that mir-193b-3p, mir-301b, mir-205-5p, mir-132-3p, mir-212-3p, mir-1271-5p, and mir-137 may contribute significantly in tobacco-mediated tumor formation. We identified 11 methylation driver genes (including *EIF5A2*, *GBP6*, *HGD*, *HS6ST1*, *ITGA5*, *NR2F2*, *PLS1*, *PPP1R18*, *PTHLH*, *SLC6A15*, and *YEATS2*), and methylation modifications of some of these genes have not been reported to be associated with tumors. We constructed a 46-gene model that predicted overall survival with good predictive power. We next drew nomograms of each cancer type. Interestingly, calibration diagrams and concordance indexes are verified that the nomograms were highly accurate for the prognosis of patients. Meanwhile, we found that the 46-gene model has good applicability to the overall survival as well as to disease-specific survival and progression-free intervals. The results of this research provide new and valuable insights for the diagnosis, treatment, and follow-up of cancer patients with different smoking histories.

## 1 Introduction

**FIGURE 1:**
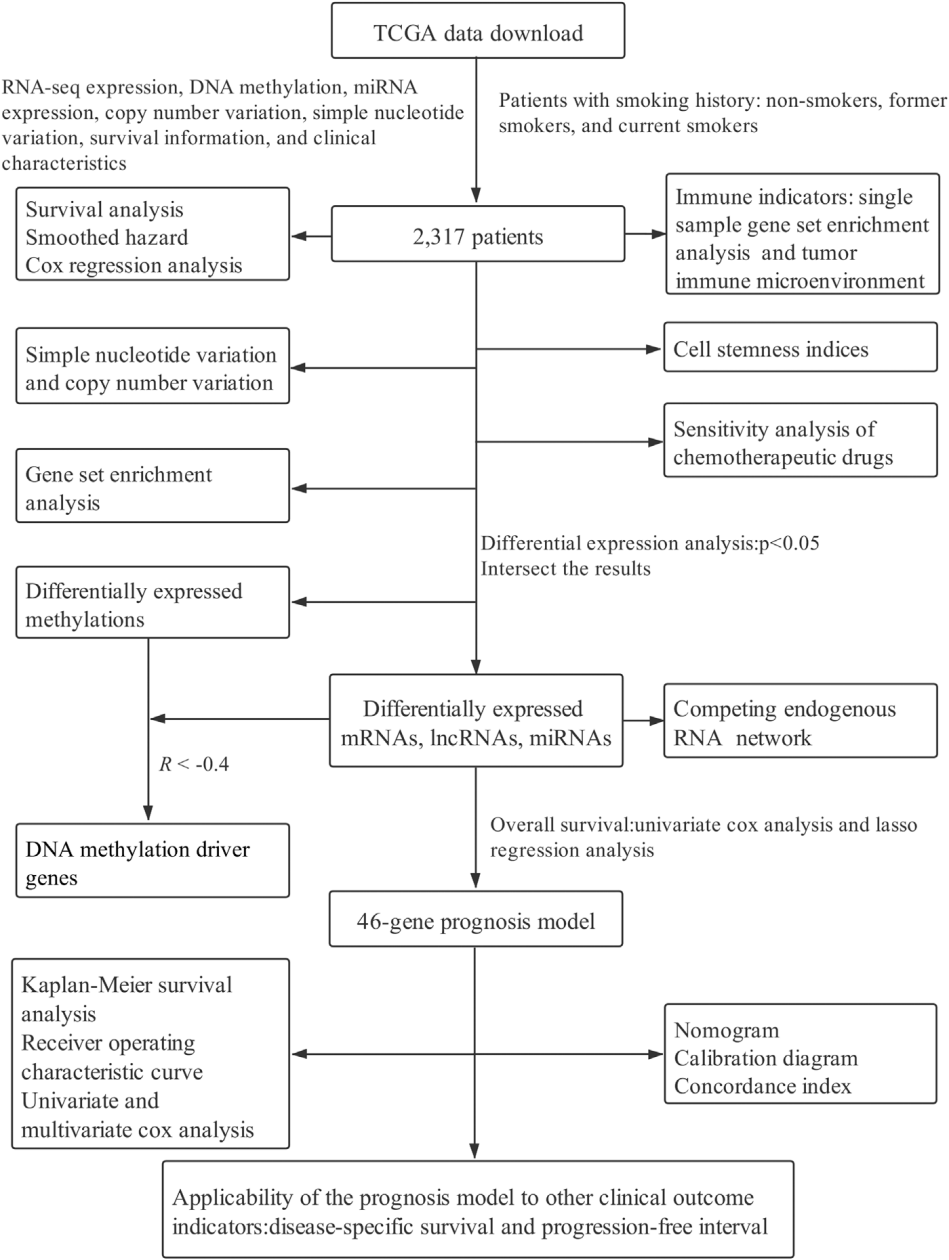
The process of this study.

Smoking is extremely harmful to human health, resulting in more cardiovascular diseases, chronic obstructive pulmonary disease, and different types of cancers (Kulhánová et al., 2020; Stang et al., 2021). It has been reported that smoking can increase the incidence of many diseases as well as lead to a poor prognosis and even increase the recurrence risk of cancer patients (Parsons et al., 2010; Rieken et al., 2015; Foerster et al., 2018). Indeed, according to the global burden of disease in 2019, smoking ranks second among the most important risk factors for death attribution in the whole population, accounting for 8.71 million people, only after high systolic blood pressure (GBDRF Collaborators, 2020). Studies have shown that smoking is detrimental not only to active smokers but also to passive smokers (Eriksen et al., 1988; Dugué et al., 2020). The carcinogenic effects of smoking mainly include the following aspects. First, the free radicals produced by smoking directly damage the cell components, leading to DNA damage and tumor formation. Second, smoking can induce mutations in a variety of genes, causing continuous proliferation of cells and malignant transformation. Third, it can cause the transformation of the inflammatory response to malignant transformation (Husgafvel-Pursiainen et al., 2000; Vähäkangas et al., 2001; Taioli, 2008; Takahashi et al., 2010). Previous studies have established that cancer patients who had smoked lived shorter compared with those who had never smoked, while smoking cessation can increase the survival time of smokers, and the earlier they quit, the longer they live (Doll et al., 2004). Elsewhere, a meta-analysis of smoking status on colorectal cancer (CRC) prognosis showed a poor overall survival (OS) rate for current smokers than non-smokers, whereas Cox regression analysis revealed that current smokers had a higher risk of poorer prognosis. In comparison, quitting smokers can improve their OS compared with current smokers, thus exhibiting a higher specific survival (Ordóñez-Mena et al., 2018). Given this background, this study integrated multiple-omics data from The Cancer Genome Atlas (TCGA) database to explore possible underlying molecular mechanisms among non-smokers, former smokers, and current smokers. A flow chart of this study is provided in Figure 1.

## 2 Methods and Materials

### 2.1 Data Downloading and Processing

Patients with a past smoking history (including non-smokers, former smokers, and current smokers) were screened from the TCGA database were included in our study, as well as downloaded their level 3 RNA-seq expression, DNA methylation (Illumina Human Methylation 450), miRNA expression, somatic copy number variations (CNVs, masked copy number segment), and simple nucleotide variations (SNVs, VarScan2 Variant Aggregation and Masking) from TCGA–GDC portal (https://portal.gdc.cancer.gov/). We also downloaded prognostic indicators such as OS, disease-specific survival (DSS), progression-free interval (PFI), as well as clinical characteristics, including gender, age, grade, and stage, among others. However, we did not include the prognostic marker disease-free interval in this study because it had too many missing values. Strawberry Perl (version: 5.30.0.1) (http://strawberryperl.com/) was used to annotate gene expression profiles. If multiple probes corresponded to one gene, the average value was taken as the expression level of the gene and expression converted by log2 ((transcripts per kilobase of exon model per million mapped reads) +1). We followed the TCGA usage rules, and thus approval from the ethics committee was not required for this work. All databases were up-to-date as of December 15, 2020.

### 2.2 Survival and Risk Analyses

Prognostic differences and the hazard ratio (HR) were evaluated among the three patients with a past smoking history with different survival indicators (OS, DSS, and PFI). We employed the Cox regression analysis to evaluate the effects of different smoking histories (non-smoker, former smoker, and current smoker were defined as 0, 1, and 2, respectively), with other clinical characteristics such as gender (females and males were defined as 0 and 1, respectively), stage and age, on the prognosis of patients.

### 2.3 Immunological Content of Patients With Different Smoking Histories

To quantify immune-related cells, functions, and pathways of each patient, we used a single-sample gene set enrichment analysis (ssGSEA), which was implemented using the R-project packages GSVA and GSEA (Barbie et al., 2009). Next, the ESTIMATE method was applied to quantify the tumor immune microenvironment, including stromal score, immune score, estimate score, and tumor purity (Yoshihara et al., 2013). Additionally, the B-cell receptor (BCR) diversity (BCR Richness, BCR Shannon), leucocyte infiltration, neoantigens, homologous recombination defects (HRD), cancer testis antigen (CTA), and intratumor heterogeneity of each patient were obtained from a study by Thorsson et al. (2018). We subsequently explored the differences in these indicators among patients with different past smoking histories.

### 2.4 Analysis of the Difference of Stemness Indices in Patients With Different Smoking Histories

The mRNA stemness indices (mRNAsi), DNA methylation stemness indices (mDNAsi), differentially methylated probes-based stemness index (DMPsi), enhancer-based stemness index (ENHsi), RNA expression-based epigenetically regulated-mRNAsi (EREG-mRNAsi), and DNA methylation-based (EREG-mDNAsi) of each patient were obtained from the study by Malta et al. (2018), in which we compared the differences in these indicators among patients characterized by different smoking histories. The stemness indices were rated between 0 and 1, signifying that the closer the stemness indices were to 1, the lower the level of tumor cell differentiation as well as the stronger the tumor cell stemness characteristics.

### 2.5 Somatic Simple Nucleotide and Copy Number Variations Analyses

The incidence of SNV events among different smoking history was compared. tumor mutation burden (TMB), usually quantified as the number of mutations per megabases, was defined as the total number of nonsynonymous mutations in each coding region of the tumor genome. CNV events (loss or gain) in each sample were integrated and analyzed using the Genomic Identification of Significant Targets in Cancer (GISTIC) 2.0 (https://cloud.genepattern.org/) (Mermel et al., 2011). The reference genome file was BSgenome.Hsapiens.UCSC.hg38, while segment mean = log_2_ (copy number/2). In particular, the value of a segment of mean greater than 0.2 was defined as gain (recorded as 1), a segment of mean with less than −0.2 was defined as loss (recorded as −1), and a segment of mean between −0.2 and 0.2 was defined as without CNV (recorded as 0). Finally, the total number of genes with CNV loss or gain at the focal or arm levels was defined as CNV loss or gain burden (Shen et al., 2019). We used R-project maftools package to visualize the results (Mayakonda et al., 2018).

### 2.6 Chemotherapeutic Response Prediction

The response to chemotherapy in each patient was predicted with the Genomics of Drug Sensitivity in Cancer (GDSC) (https://www.cancerrxgene.org/). The R-project prophet package was used to perform the prediction, while the half-maximal inhibitory concentration (IC50) of each patient was predicted through ridge regression. Based on the GDSC training set, the precision of prediction was verified by 10 cross-validations (Geeleher et al., 2014).

### 2.7 Pathways Enrichment of Patients With Different Smoking Histories

Gene Set Enrichment Analysis (GSEA) was applied to identify the enrichment of oncogenic signature in patients with different smoking histories, which was performed using the GSEA software (http://software.broadinstitute.org/gsea/downloads.jsp) (version: 4.0.3). The criteria for enrichment difference included: |normalized enrichment score (NES)| > 1, the nominal *p*-value (NOM *p*-value) < 0.05, and the false discovery rate *q*-value (FDR *q*-value) ≤ 0.25.

### 2.8 Identification of Differentially Expressed Genes and Construction of Competing Endogenous RNA Regulation Network

The R-project package edgeR was used to screen the differentially expressed mRNAs (DEmRNAs), differentially expressed lncRNAs (DElncRNAs), and differentially expressed miRNAs (DEmiRNAs), according to the screening criteria *p*-value < 0.05 (Robinson et al., 2010). Afterward, the competing endogenous RNA (ceRNA) network was established using DEmRNAs, DElncRNAs, and DEmiRNAs. LncRNA-miRNA links were estimated by the use of miRcode (http://www.mircode.org/), while the target genes of miRNAs were predicted with miRDB (version:6.0) (http://www.mirdb.org/), mirTarBase (version:8.0) (https://mirtarbase.cuhk.edu.cn/~miRTarBase/miRTarBase_2022/php/index.php), and TargetScan (version:7.2) (http://www.targetscan.org/vert_72/). We defined the predicted target genes in at least two databases. Next, we used Cytoscape (version:3.8.1) (https://cytoscape.org/) to visualize the ceRNA network.

### 2.9 DNA Methylation Driver Gene Analyses

Differentially expressed methylated genes between any two of the three smoking histories were screened according to the screening criteria *p*-value < 0.05. Spearman correlation analysis was applied to calculate the correlation coefficient (*R*) between methylation level and its mRNA expression. A *R* < -0.4 and *p* < 0.05 was considered as a DNA methylation driver gene.

### 2.10 Establishing and Assessing a Smoking-Related Prognostic Model for Patients

Univariate Cox analysis was used to identify differentially expressed genes associated with OS of patients with *p*-value < 0.05. The R-project glmnet package was used for lasso regression analysis to prevent the over fitting of the model (alpha = 1) (Friedman et al., 2010). Upon constructing a multivariate Cox proportional hazards regression model, we obtained the risk score of each sample using the regression coefficient and expression of the gene as follows: Risk score = $\overset{\text{n}}{\underset{\text{i}=1}{\sum }}\mathrm{Coefficient (gen}e_{i}\mathrm{)*expression (gen}e_{i})$ .

Moreover, we used R-project survival and survminer packages to examine the difference in OS between different risk score groups. Then, the Cox regression analysis was employed to assess the effects of the model along with other clinical characteristics on OS. The R-project survival ROC package was used to draw the receiver operating characteristic curve (ROC) as well as calculate the area under the curve (AUC) to determine the predictive ability of the model (Heagerty et al., 2000).

Nomogram was established to diagnose or predict the progression or prognosis of the disease by combining multiple clinical indicators. Harrell’s concordance index (C-index) and calibration diagram were used to estimate the consistency between the predictive results of the nomogram and the actual occurrence of events. Further, R-project rms, survival, and survcomp packages were used to draw the calibration diagram and calculate the C-index (Schröder et al., 2011). Lastly, we assessed the applicability of the model to other clinical outcome indicators (DSS and PFI).

### 2.11 Statistical Analysis

The R-project (version: 3.6.3) (https://www.r-project.org/) and bio conductor packages (http://bioconductor.org/) were used for statistical analysis and visualization. Then, χ^2^ and *p*-values were calculated using the Chi-square or Fisher’s exact tests. The linear correlation between the two variables was analyzed using the Spearman correlation coefficient. Differences between the two sets were compared using the Wilcoxon rank-sum test, while those between multiple groups were compared using Kruskal–Wallis test. All statistical tests were two-sided and a value of *p* < 0.05 was considered statistically significant. “*,” “**,” “***” and “ns” indicates *p* < 0.05, *p* < 0.01, *p* < 0.001, and *p* > = 0.05, respectively.

## 3 Results

### 3.1 Smoking Exerts a Negative Effect on the Prognosis of Patients

**FIGURE 2:**
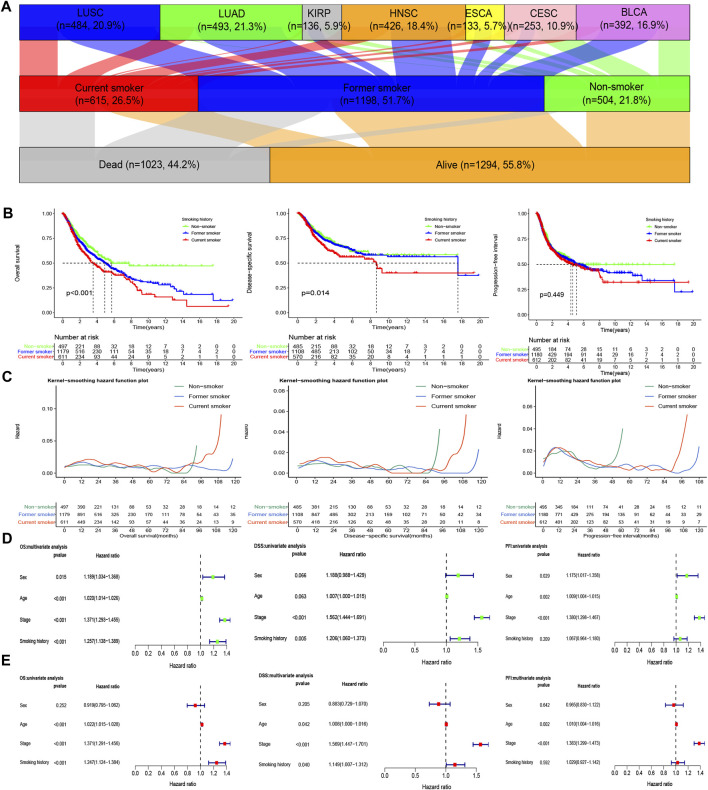
Influence of different smoking histories on the prognosis indicators of cancer patients. **(A)** The Sankey diagram of cancer type, smoking history, and the survival status of the included samples. **(B)** KM curves showed differences in OS, DSS, and PFI among patients with different smoking histories. **(C)** The smoothed hazard estimates of OS, DSS, and PFI in different smoking history patients. **(D,E)** The Cox regression analysis evaluated the influence of different smoking histories on OS, DSS, and PFI along with other clinical features.

In this study, we collected 2,317 patients with various smoking histories from seven cancer types, namely, bladder urothelial carcinoma (BLCA), cervical squamous cell carcinoma and endocervical adenocarcinoma (CESC), esophageal carcinoma (ESCA), head and neck squamous cell carcinoma (HNSC), kidney renal papillary cell carcinoma (KIRP), lung adenocarcinoma (LUAD), and lung squamous cell carcinoma (LUSC). The detailed information is summarized in Supplementary Table S1. The Sankey diagram of cancer type, smoking history, and survival status was depicted in Figure 2A.

The results of survival analysis of patients with different smoking histories revealed that non-smokers enumerated the best OS and DSS, followed by former smokers, while on the other hand, current smokers exhibited the worst OS and DSS (*p* < 0.05). Besides, current smokers also have a higher risk for poor OS and DSS, followed by former smokers, whereas non-smokers have a lower risk (Figures 2B,C).

For PFI, we observed no significant difference (*p* > 0.05) among patients with different smoking histories, but the 10- ,15-years PFI was the highest for non-smokers, followed by former smokers, while that of the current smokers was the lowest. However, there was no statistical difference in the risk of PFI among patients with different smoking histories (Figures 2B,C).

The Cox regression analysis results demonstrated that smoking history was an independent factor for OS and DSS in patients. Notably, current smoking was an independent risk factor for OS and DSS. Nevertheless, the effect of smoking history on PFI was not statistically different (Figures 2D,E).

### 3.2 Differences in Immune Indicators Among Patients With Different Smoking Histories

**FIGURE 3:**
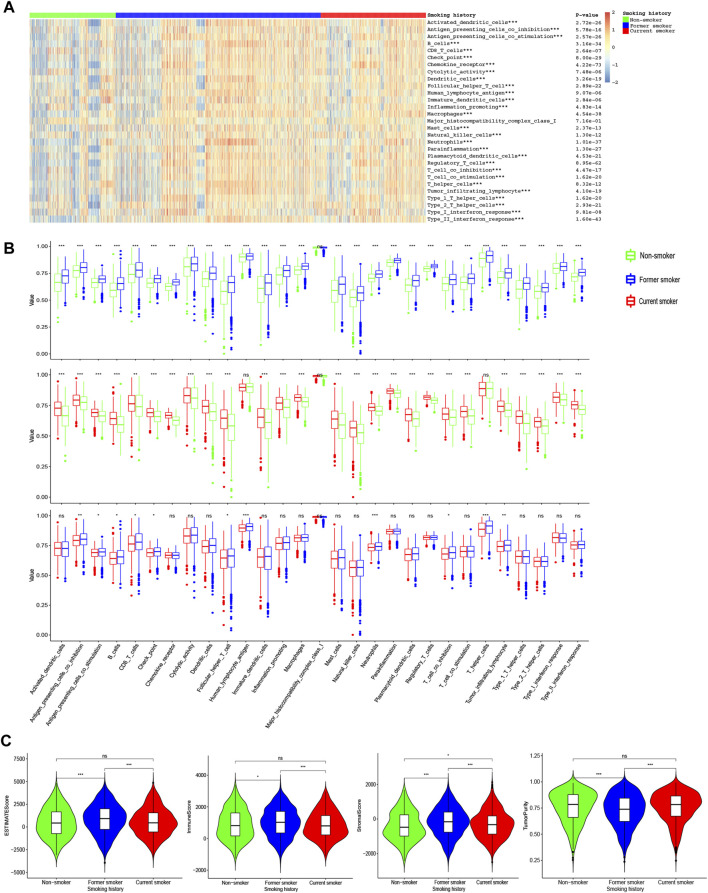
ssGSEA of patients with different smoking histories. **(A)** The landscape of 29 immune indicators among patients with different smoking histories. **(B)** Differences in 29 immune indicators among patients with different smoking histories. **(C)** Differences in the stromal score, immune score, estimate score, and tumor purity among patients with different smoking histories.

We found higher immune levels as well as lower tumor purity in former smokers (Figure 3). Antigen-presenting cell co-inhibition, antigen-presenting cell co-stimulation, B cells, CD8^+^ T cells, checkpoint, follicular helper T cells, natural killer (NK) cells, T cell co-inhibition, and tumor-infiltrating lymphocyte were the highest in former smokers, lowest in current smokers, and moderate in non-smokers. In addition, the levels of dendritic cells (DCs), chemokine receptor (CCR), cytolytic activity, immature dendritic cells, inflammation-promoting, macrophages, mast cells, neutrophils, para inflammation, plasmacytoid dendritic cells, regulatory T cells, T cell co-stimulation, type 1 T helper cells, type 2 T helper cells, type I interferon (IFN) response, type II IFN response was higher in former smokers and current smokers than those in non-smokers, while there was no difference between former smokers and current smokers. The levels of the immune score and ESTIMATE score were higher in former smokers than those in non-smokers and current smokers, while there was no difference between non-smokers and current smokers. The stromal score was the highest in former smokers, followed by current smokers, and the lowest in non-smokers.

**FIGURE 4:**
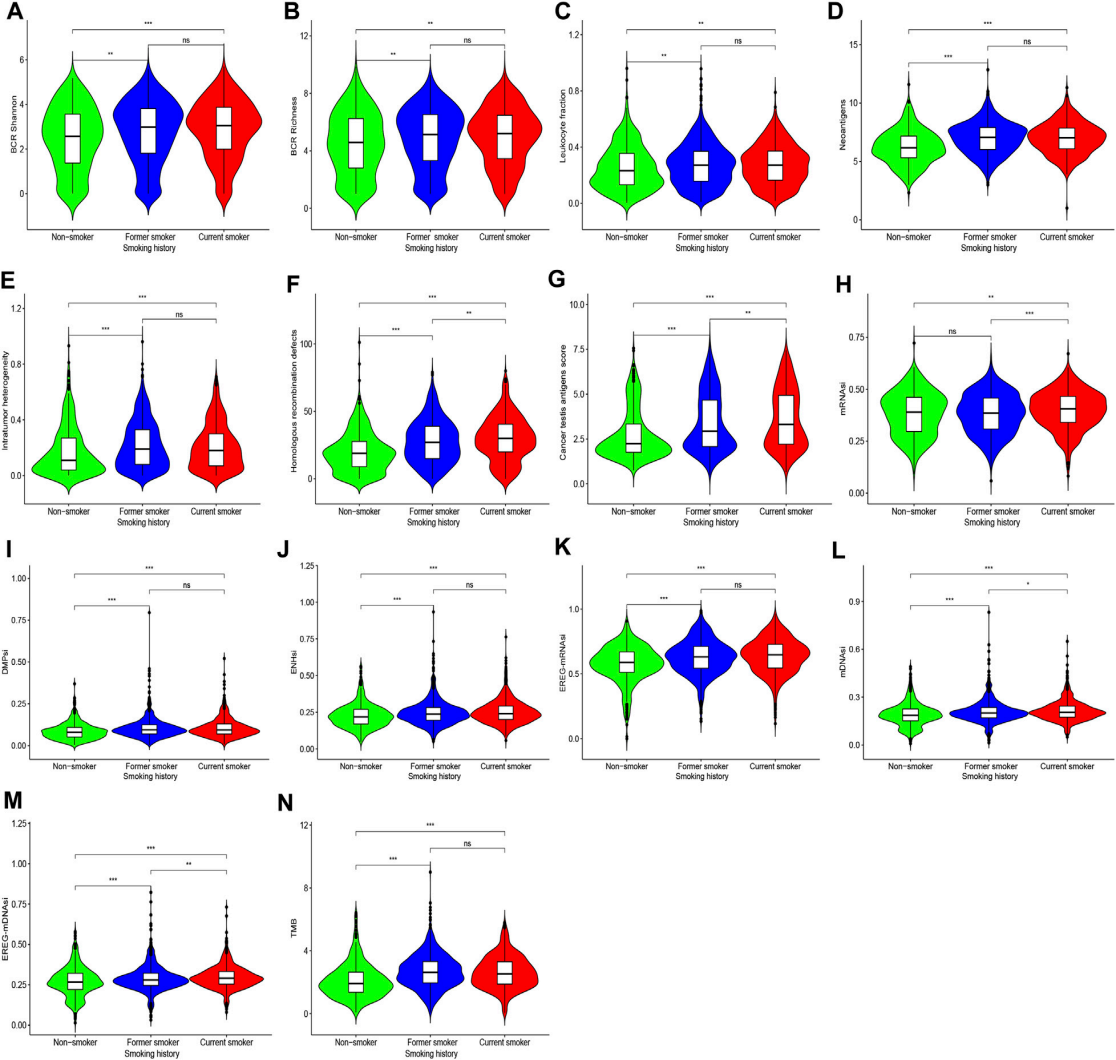
Shows differences in BCR diversity [**(A)** BCR Shannon, **(B)** BCR Richness], **(C)** leukocyte fraction, **(D)** neoantigens, **(E)** intratumoral heterogeneity, **(F)** HDR, **(G)** CTA scores, **(H)** mRNAsi, **(I)** DMPsi, **(J)** ENHsi, **(K)** EREG-mRNAsi, **(L)** mDNAsi, **(M)** EREG-mDNAsi, and **(N)** TMB among patients with different smoking histories.

For BCR diversity (BCR Shannon and BCR Richness), leukocyte fraction, neoantigens, and intratumoral heterogeneity, we identified that smokers were markedly higher than non-smokers, but there was no significant difference between former smokers and current smokers. Next, we observed that the HRD and CTA scores were the highest in current smokers, followed by former smokers, and lowest in non-smokers (Figures 4A–G).

### 3.3 Smoking can Induce Tumor Cell Stemness Formation

Compared with former smokers and non-smokers, current smokers exhibited higher mRNAsi, and there was no difference between former smokers and non-smokers. For DMPsi, ENHsi, and EREG-mRNAsi, smokers were considerably higher compared with non-smokers. In addition, mDNAsi and EREG-mDNAsi were the highest in current smokers, followed by former smokers, and the lowest in non-smokers. These findings indicate that the stimulation of smoking can induce tumor cell stemness formation, which may be reversed following smoking cessation in some indicators (Figures 4H–M).

### 3.4 Smoking Induces More Somatic Mutations and Copy Number Variations That Remain the Same After Smoking Cessation

**FIGURE 5:**
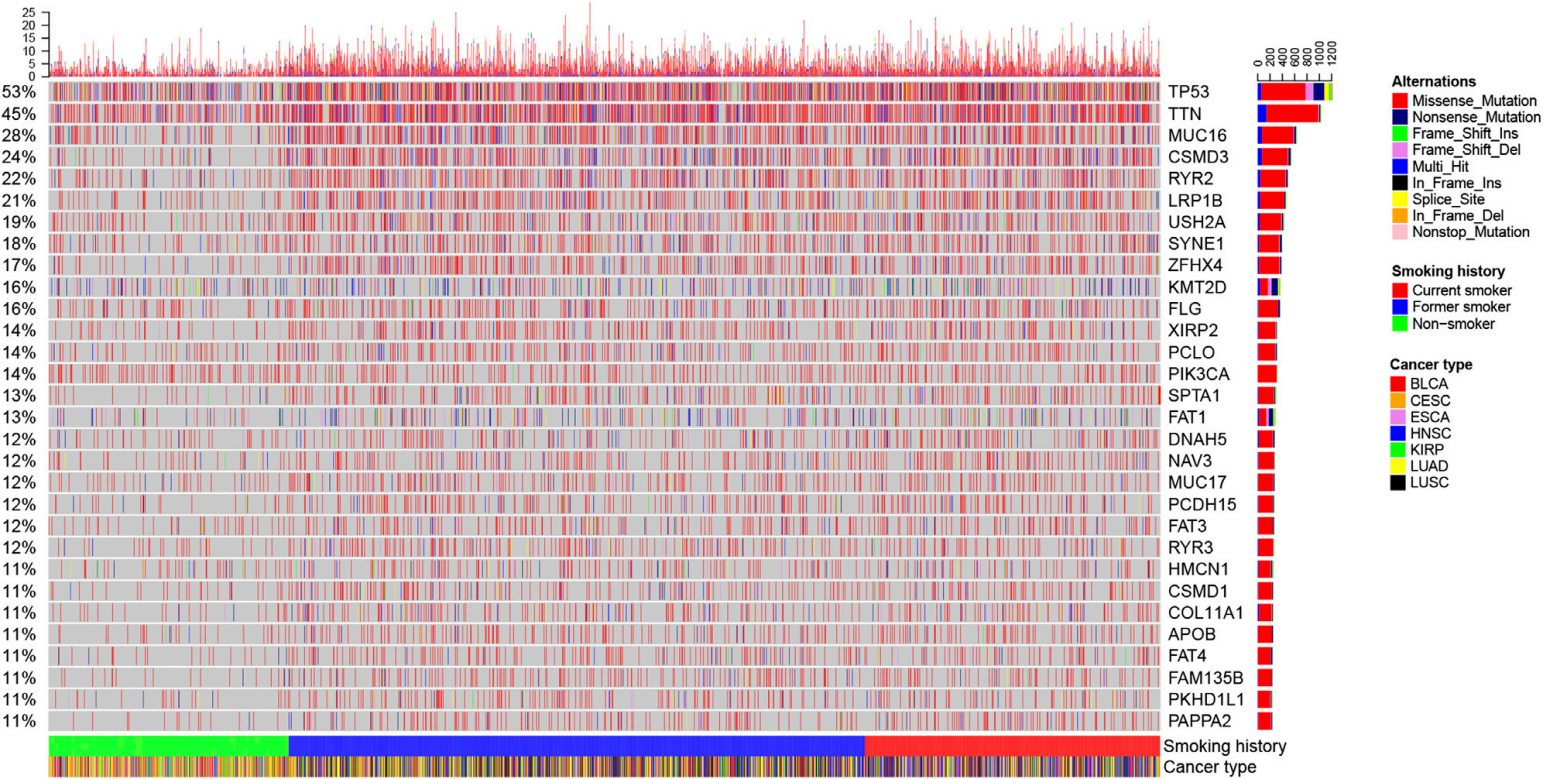
Landscape of the top 30 genes with higher SNV incidence in patients with different smoking histories.

We herein recorded that the TMB of non-smokers was lower than that of smokers, but there was no difference in TMB between former smokers and current smokers (Figure 4N). Figure 5 and Supplementary Figure S1A shows the genes with higher SNV incidence in patients with different smoking histories. Importantly, the SNV incidence of multiple genes, including *TP53*, *TTN*, *MUC16*, *CSMD3*, *RYR2*, *LRP1B*, *USH2A*, *SYNE1*, *ZFHX4*, *FLG*, *XIRP2*, and *PCLO*, among others, in smokers were markedly increased compared with non-smokers. Except for *DNAH5* and *NAV3*, there was no statistical difference in SNV incidence of other genes between current smokers and former smokers.

**FIGURE 6:**
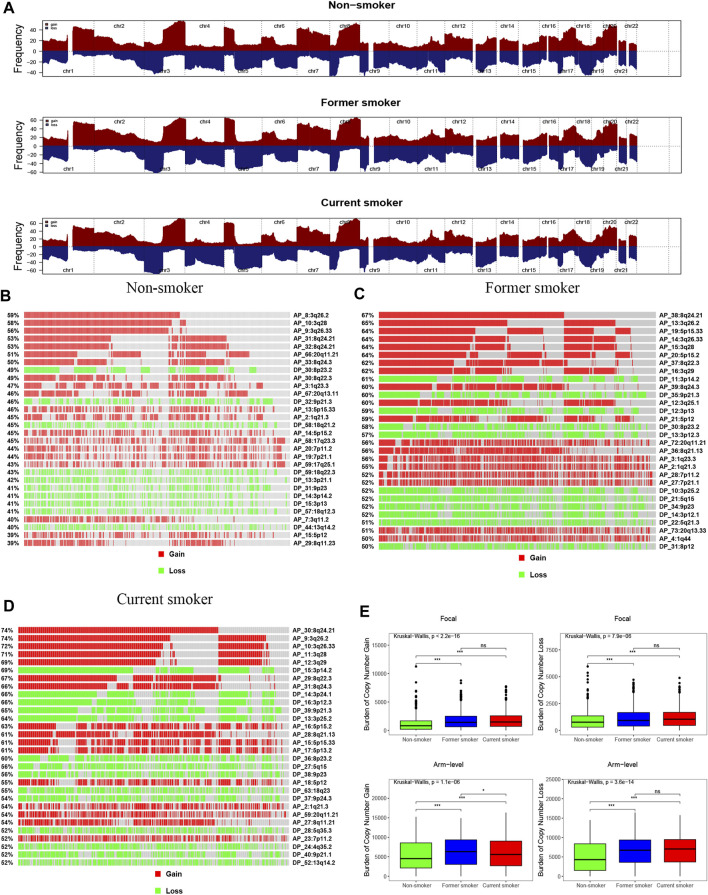
Copy number profiles of patients with different smoking histories. **(A)** Gene fragments are placed on chromosomes ranging from chromosome 1 to 22, in which gain is dark red while loss is dark blue. Differences in the gene fragment distribution of CNV events in patients with different smoking histories [**(B)**: non-smoker, **(C)**: former smoker, **(D)**: current smoker]. **(E)** Differences in CNV gain and loss burden at focal and arm in patients with different smoking histories.

Our results, based on GISTIC2.0, revealed that chromosomes 3, 8, 1, 5, 17, and 20 exhibited a higher incidence of CNV gain events, while chromosomes 8, 9, 19, 17, 3, 4, and 5 had a higher incidence of CNV loss events (Figure 6A). Figure 6 presents the fragment for the most frequent CNV events among patients with different smoking histories. We identified that the CNV gain of non-smokers occurred primarily in 3q26.2, 3q28, 3q26.33, 8q24.21, 8q24.21, 20q11.21, and 8q24.3, while loss occurred mainly in 8p23.2, 9p21.3, and 18q21.2 (Figure 6B). For former smokers, CNV gain occurred predominately in 8q24.21, 3q26.2, 5p15.33, 3q26.33, 3q28, 5p15.2, and 8q22.3, whereas CNV loss occurred mainly in 3p14.2, 9p21.3, and 3p13 (Figure 6C). For current smokers, CNV gain prevalently occurred in 8q24.21, 3q26.2, 3q26.33, 3q28, 3q29, and 3p14.2, while loss occurred primarily in 3p14.2, 3p24.1, 3p12.3, 9p21.3, 3p25.2, and 8p23.2 (Figure 6D). Upon many chromosome segments, such as 8q24.21, 3q26.2, 3q26.33, and 8q22.3, current smokers displayed the highest incidence of CNV events, followed by former smokers, while non-smokers had the lowest.

In comparison, non-smokers have a lower burden of gain and loss at the focal and arm levels than smokers. For arm level gain burden, the level of former smokers was higher when compared with that of current smokers. Nonetheless, there were no statistical differences in the focal level gain and loss burden, and the arm level loss burden, between former smokers and current smokers (Figure 6E).

**FIGURE 7:**
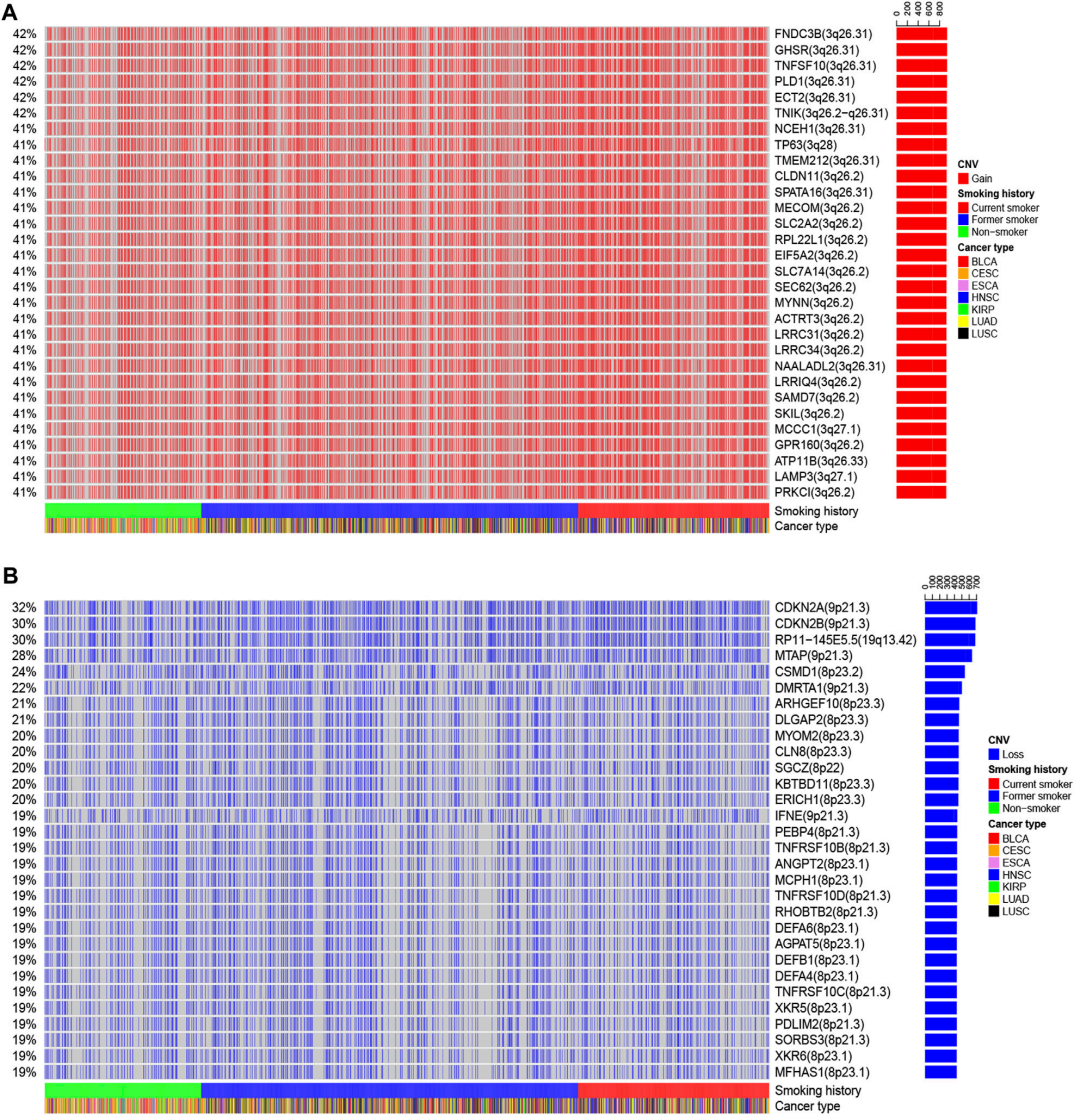
The landscape of the top 30 genes in the incidence of **(A)** CNV gain and (B) CNV loss in patients with different smoking histories.

We also identified the top 30 genes characterized by a higher incidence of CNV events (Figure 7 and Supplementary Figures S1B,C). The CNV gain predominantly occurred in *FNDC3B* (3q26.31), *GHSR* (3q26.31), *TNFSF10* (3q26.31), *PLD1* (3q26.31), *ECT2* (3q26.31), *TNIK* (3q26.2−q26.31), *NCEH1* (3q26.31), *TP63* (3q28), and SLC2A2 (3q26.2) (Figure 7A). On the other hand, CNV loss mainly occurred in *CDKN2A* (9p21.3), *CDKN2B* (9p21.3), *RP11−145E5.5* (19q13.42), *MTAP* (9p21.3), *CSMD1* (8p23.2), *DMRTA1* (9p21.3), *ARHGEF10* (8p23.3), *DLGAP2* (8p23.3), *MYOM2* (8p23.3), *CLN8* (8p23.3), and *KBTBD11* (8p23.3) (Figure 7B).

We observed that the incidence of the multiple gene CNV gain was the highest in current smokers, followed by former smokers, and the lowest in non-smokers (Supplementary Figure S1B). In terms of CNV loss, the incidence of multiple genes, including *CDKN2A*, *CDKN2B*, *RP11-145E5.5*, *MTAP*, and *DMRTA1*, was the highest in current smokers, followed by former smokers, and lowest in non-smokers (Supplementary Figure S1C), suggesting that smoking cessation could reverse CNV events in some genes.

### 3.5 Chemotherapeutic Response Prediction

**FIGURE 8:**
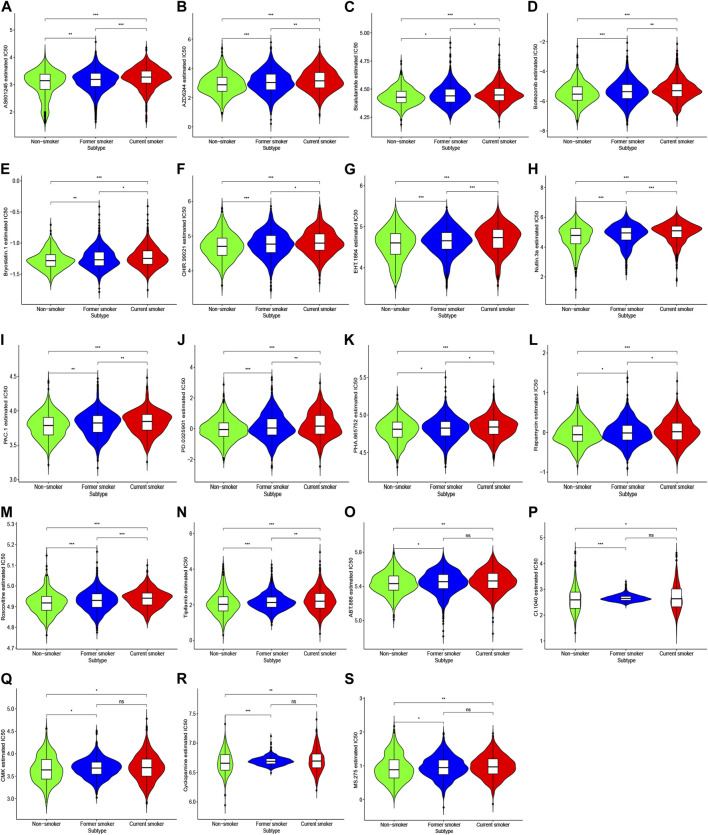
IC50 of response to chemotherapeutic drugs in patients with different smoking histories. Red, blue, and green represent current smokers, former smokers, and non-smokers, respectively.

Among a variety of small molecule compounds, including AS601245 (JNK inhibitor), AZD6244 (MEK inhibitor), bicalutamide (androgen receptor competitor), bortezomib (proteasome inhibitor), bryostatin.1 (protein kinase C activator), CHIR.99021 (glycogen synthase kinase-3 selective inhibitor), EHT.1864 (Rac family small GTPases inhibitor), Nutlin.3a (Mam2 inhibitor), PAC.1 (procaspase-3 activator), PD.0325901 (MEK inhibitor), PHA.665752 (c-Met kinase inhibitor), rapamycin (mTOR inhibitor), roscovitine (cyclin-dependent kinase inhibitor), and tipifarnib (farnesyltransferase inhibitor), current smokers depicted the highest IC50, followed by former smokers, whereas non-smokers exhibited the lowest IC50 (Figures 8A–N).

For ABT.888 (poly ADP-ribose polymerase inhibitor), CI.1040 (MEK inhibitor), CMK (ribosomal s6 kinase-2 inhibitor), cyclopamine (hedgehog pathway antagonist), and MS.275 (histone deacetylase inhibitor), IC50 was largely reduced in non-smokers compared with smokers. However, we noted no substantial difference between former smokers and current smokers (Figures 8O–S).

### 3.6 Gene Set Enrichment Analysis

For oncogenic signatures, VEGF A UP. V1 DN, E2F3 UP. V1 DN, and MTOR UP. V1 UP were significantly enriched in smokers than non-smokers. These pathways were also largely enriched in current smokers relative to reformer smokers (Supplementary Table S2, Sheet 1).

### 3.7 Construction of a ceRNA Network

**FIGURE 9:**
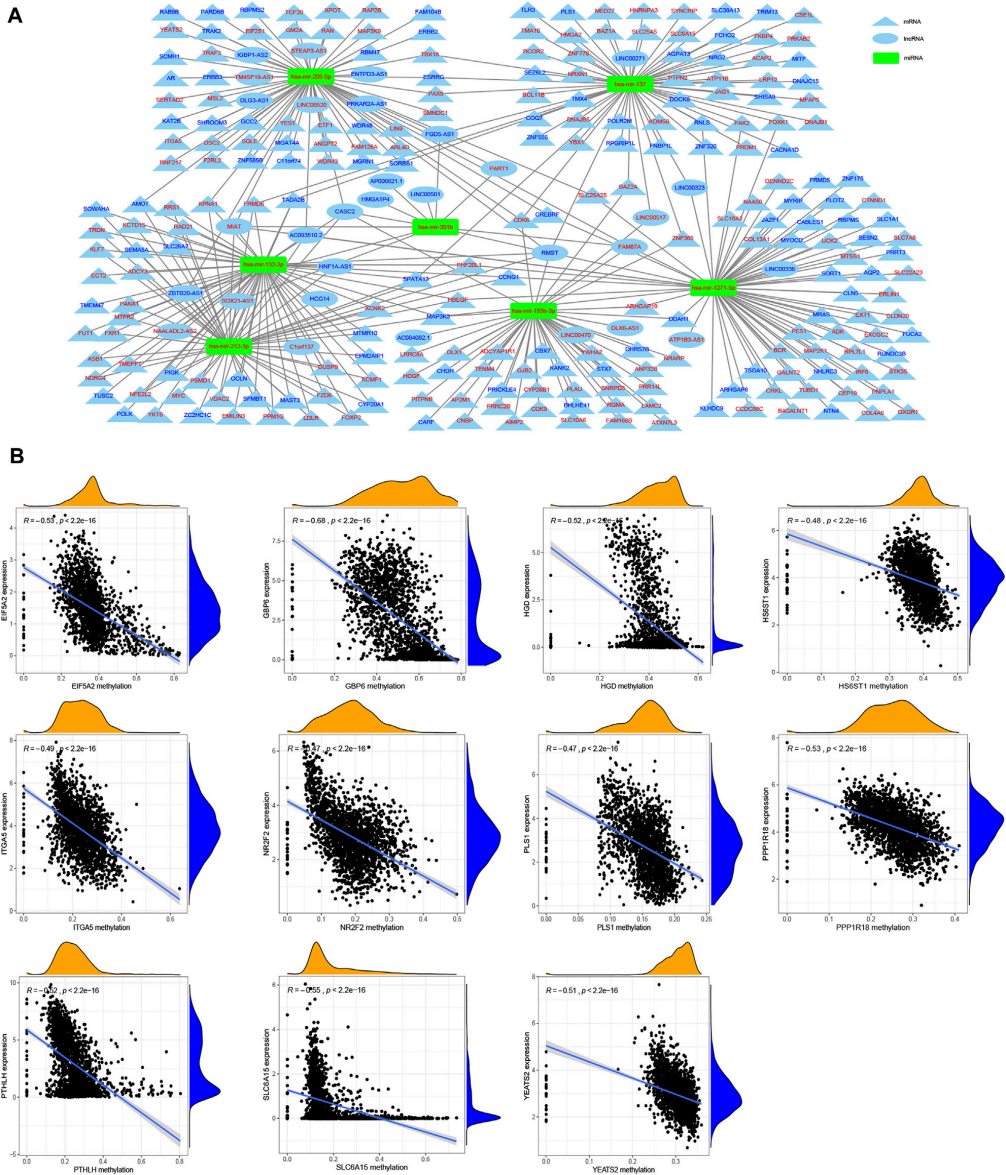
Construction of ceRNA network and identification of methylation driver genes. **(A)** CeRNA network shows the associations between the targeted lncRNAs (oval) and mRNAs (triangle) of miRNAs (rectangular) related to the tumor pathogenesis of smoking, in which red denotes up-regulation while blue indicates down-regulation. **(B)** Correlation analysis of gene expression and its methylation level.

We further screened DEGs between patients with different past smoking histories, that is, non-smokers vs. former smokers, non-smokers vs current smokers, and former smokers vs. current smokers (Supplementary Table S2, Sheet 2). After integrating the above results, we obtained 1956 DEmRNAs (1,064 upregulated and 892 downregulated), 507 DElncRNAs (233 upregulated and 274 downregulated), and 80 DEmiRNAs (74 upregulated and 6 downregulated) (Supplementary Table S2, Sheet 3). Furthermore, CeRNA network indicated that mir-193b-3p, mir-301b, mir-205-5p, mir-132-3p, mir-212-3p, mir-1271-5p, and mir-137 may play an indispensable role in tobacco-related tumor formation (Figure 9A).

### 3.8 Multiple DNA Methylation Drivers Genes Have Been Identified Associated With Smoking

Here, we identified 67 up-regulated and 416 down-regulated differentially expressed methylated genes (Supplementary Table S2, Sheet 4). Using the Spearman correlation analysis, we obtained 11 methylation driver genes, including *EIF5A2* (*R* = −0.53), *GBP6* (*R* = −0.68), *HGD* (*R* = −0.52), *HS6ST1* (*R* = −0.48), *ITGA5* (*R* = −0.49), *NR2F2* (*R* = −0.47), *PLS1* (*R* = −0.47), *PPP1R18* (*R* = −0.53), *PTHLH* (*R* = −0.52), *SLC6A15* (*R* = −0.55), and *YEATS2* (*R* = −0.51) (Figure 9B).

### 3.9 Establishment and Validation of a Smoking-Related Prognostic Model

**FIGURE 10:**
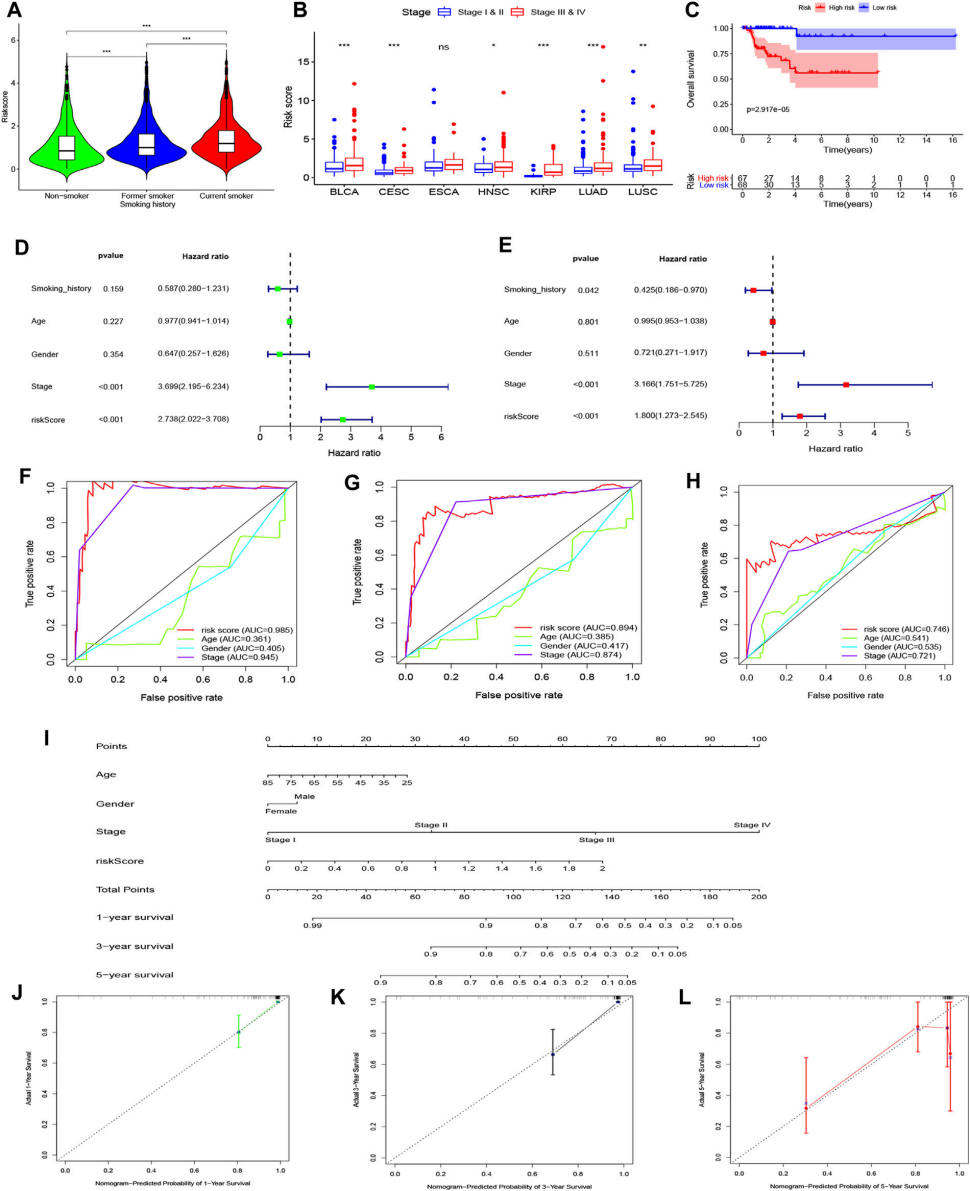
Establishment and validation of a 46-genes prognostic model for KIRP patient’s OS. **(A)** Differences in risk scores among all patients with different smoking histories. **(B)** Differences in risk scores among patients with different stages. **(C)** KM curves showed a difference in OS among KIRP patients with different risk scores. **(D)** Univariate and **(E)** multivariate Cox regression analyses were applied to analyze the effects of risk scores on OS along with other clinical features. The ROC curves of the ability of the smoking-related model and other clinical features to predict KIRP patient’s **(F)** 1-, **(G)** 3- and **(H)** 5-years OS. **(I)** A nomogram was constructed by combining the smoking-related model with age, gender, and stage to predict 1-, 3- and 5-years OS of KIRP. Calibration diagrams were applied to assess the consistency of nomogram prediction of KIRP’s **(J)** 1-, **(K)** 3- and **(L)** 5-years OS with real results.

Using the univariate Cox regression analysis, we found that 1,404 genes were associated with OS (Supplementary Table S2, Sheet 5). As a result, a 46-gene smoking-related prognostic model was constructed (the 46 genes included in the smoking-related prognostic model are outlined in Table 1). Risk scores for all samples obtained via the prognostic model are detailed in Supplementary Table S2, Sheet 6. Afterward, we uncovered that current smokers have the highest risk scores, lowest in non-smokers, and between the two were former smokers (Figure 10A). Patients with poor staging (stage III and IV) had higher risk scores compared with patients with better staging (stage I and II) for all cancer types except for ESCA (Figure 10B). Moreover, KM curves demonstrated that the OS of patients in the high-risk score group was worse compared with the low-risk score group in each cancer type (Figure 10C and Supplementary Figure S2). It is to be noted, the independent prognostic analysis elucidated that the 46-gene smoking-related model was an independent risk factor for the OS of patients in each cancer type (Figures 10D,E and Supplementary Figure S2). More interestingly, ROC shows that the 46-gene smoking-related model exhibited effective predictive power for 1-, 3-, and 5-year OS of patients in each cancer type. Furthermore, compared with other clinical indicators, the 46-gene smoking-related model possesses the superior predictive ability for the OS (Figures 10F–H and Supplementary Figure S3). To predict the OS for each type of cancer, we then constructed nomograms by combining the 46-gene smoking-related model with other clinical characteristics, including age, sex, stage, and so on (Figure 10I and Supplementary Figure S4). Both the calibration diagrams (Figures 10J–L and Supplementary Figure S4) and C-indexes (Table 2) showed that the nomograms have better predictive abilities.

**TABLE 1:** The regression coefficient of 46 genes in the 46-gene prognostic model.

Gene	Coefficient	Gene	Coefficient
GABRA3	0.071003831	HSF1	0.214233106
C10orf90	0.337893216	AC022382.1	−0.447575475
CRB3	−0.144275548	MYOCD	0.237116989
ZNF684	−0.351124244	USP4	−0.230517617
CRYZ	0.160012324	AC113346.1	−0.160210204
CHST5	0.528328195	NOP58	0.1158533
ZNF44	−0.123742252	AP000487.1	0.252927534
CYTL1	0.128171571	SCRG1	0.285617483
KRT77	0.152914276	LRP4−AS1	0.410745213
STARD4	0.14767866	LINC00539	−0.668719512
ANGPT2	0.147853794	ZCWPW1	−0.124585536
MYL2	0.077509337	KRT79	0.070985841
CXCL8	0.058884281	AC021016.2	−0.152691067
PDK2	−0.12905015	TTBK1	−0.46897621
SLCO1B1	0.2525273	RUNDC3B	−0.530468579
CCDC88C	−0.170320954	AC127521.1	0.383902578
PTCSC2	−0.168894593	TYRO3	0.15695226
BCR	−0.215305015	COL17A1	−0.04768134
SRP68	0.26087884	KRT82	−2.734361081
MARK2	0.325791745	NOS1	0.110625126
TUBD1	−0.198357131	MFHAS1	−0.171772138
KBTBD2	0.178448729	AC015908.3	−0.356104779
MIR193BHG	0.142822324	VAX1	0.215776961

**TABLE 2:** The C-index of the nomograms was used to predict the prognostic indicators in each cancer type.

Cancer type	OS	DSS	PFI
BLCA	0.728	0.745	0.683
CESC	0.748	0.789	0.726
ESCA	0.784	0.819	0.653
HNSC	0.700	0.732	0.668
KIRP	0.906	0.934	0.868
LUAD	0.726	0.746	0.658
LUSC	0.639	0.718	0.662

Next, we explored the applicability of the 46-gene smoking-related model for DSS and PFI. Our findings demonstrated that the model has good applicability for other cancer types except for ESCA. Patients with high-risk scores displayed poor DSS and PFI, while risk scores were independent risk factors for DSS and PFI (Supplementary Figures S5, S6). Remarkably, ROC analysis shows that the 46-gene smoking-related model had better predictive power for 1-, 3-, and 5-year DSS and PFI of patients in each cancer type (Supplementary Figure S7). Similarly, we constructed nomograms to predict the DSS and PFI of patients. The calibration diagrams and C-indexes show that the nomograms have high accuracy (Supplementary Figures S8, S9).

## 4 Discussion

Smoking has been shown to cause the occurrence of a variety of diseases. Currently, there is a dearth of information regarding non-smokers, former smokers, and current smokers. In this present investigation, we explored the underlying possible molecular mechanisms of smoking history on the occurrence and development of tumors based on multi-omics analysis of smoking-related tumors in the TCGA database. We uncovered that smoking history exerted an effect on the prognosis of cancer patients. Additionally, patients with different smoking histories showed differences in immune content, tumor cell stemness, genome stability, and sensitivity to chemotherapy drugs. Multiple miRNAs that may be associated with the pathogenesis of smoking-related tumors were identified. We also built a smoking-related model to predict the prognosis of patients, which was characterized by high accuracy and wide clinical applicability.

We studied the difference in prognosis among patients with different smoking histories. The results showed that non-smokers had better OS and DSS than smokers. Importantly, quitting smoking could improve the prognosis and prolong survival time. Smoothed hazard estimates demonstrated that current smokers exhibited the highest risk for poor OS and DSS, followed by former smokers, while non-smokers had the lowest risk. Notably, the Cox regression analysis identified that current smoking was an independent risk factor for OS and DSS, which agrees with previous research results that, compared with non-smokers, smokers have a poor prognosis. Also, smoking cessation can improves the prognosis of smokers, whereas smoking history was an independent prognostic factor for cancer patients (Vineis et al., 1988; Manjer et al., 2000; Sardari Nia et al., 2005; Tsao et al., 2006; Zhou et al., 2006; Vladimirov and Schiodt, 2009; Kenfield et al., 2011).

It has been reported that smoking can lead to changes in innate and acquired immune systems, as well as changes in the number and function of immune indicators, promote the production of a variety of pro-inflammatory factors, and inhibit the production of anti-inflammatory factors (Arnson et al., 2010). In terms of leukocyte infiltration, intratumoral heterogeneity, and neoantigens, smokers were significantly higher relative to non-smokers. Therefore, it is apparent that smoking cessation does not lead to any immediate change in this status. This is probably because tobacco can induce more leukocyte infiltration as well as neoantigens in the body (Inamura et al., 2017; Ahmad et al., 2019). Another reason for drug resistance is intratumor heterogeneity, which can be caused by tobacco (Salk et al., 2010; Alexandrov et al., 2016). Through ssGSEA, we found considerable differences in multiple immune indicators among patients with different smoking histories. Inflammation and immune regulation due to smoking are potentially important mechanisms in the development of cancer. Tobacco smoke contains a wide variety of mutagenic and carcinogenic compounds, including carbon monoxide, nicotine, nitrogen oxides, and cadmium, among others. Smoking has been established to cause many systemic immune changes, changes in the number of macrophages, neutrophils, eosinophils, mast cells, and DCs, as well as alterations in the function of macrophages and neutrophils (Shiels et al., 2014). Li et al. (2018) found that the abnormal activation of mast cells plays a vital role in abnormal pulmonary immune function caused by smoking, which may lead to tumorigenesis and development. Smoking may increase inflammation by increasing the number and function of DCs and can lead to a sharp increase in the number of DCs and Langerhans cells (Soler et al., 1989). Smoking can upregulate the expression of CCR7 and CD86, and significantly promote the transport and response of DCs in the airway of mice to promote allergic airway inflammation (Robays et al., 2009). Tobacco did not induce inflammation or immune response in CD8 knockout mice (Maeno et al., 2007). The IFN-γ-inducible protein-10 derived from CD8^+^ T cells promotes the production of elastin in macrophages, leading to elastin fragmentation and lung damage. In addition to activating the expression of CD8+T cells, cigarette smoke also induces CD8^+^ T cells to produce more toll-like receptor proteins and thereby increasing the expression of cytokines (Nadigel et al., 2011). Current smokers have a significantly higher risk of acute prostatitis than former smokers and non-smokers (Moreira et al., 2015), and smoking can induce B-cell signatures of prostatitis and prostate cancer in current smokers, leading to immunoglobulin expression (Prueitt et al., 2016). Tobacco exposure increased the expression of IFN-γ and CD107a in the NK cells of mice and enhanced the NK cells response (Motz et al., 2010). Cigarette smoke also caused mouse NK cells to express more T helper cell-17 cytokine (Bozinovski et al., 2015). Levels of a variety of immune indicators were higher in former smokers than current smokers, possibly due to continuous tobacco stimulation resulting in a decrease in immune function, which can be reversed after quitting smoking (Cui and Li, 2010).

In this work, we found that smokers presented higher tumor cells stemness relative to non-smokers. Tobacco is well known to promote tumor resilience through the Akt-mediated ABCG2 activity to increase the proportion of lung cancer and HNSC tumor stem-like cells (An et al., 2012). Besides, it can also trigger the activation of the Sonic hedgehog pathway, which contributes significantly to the maintenance of stemness in kidney cancer and BLCA cells (Qian et al., 2018; Sun et al., 2020). Nicotine induces the expression of the embryonic stem cell factor SOX2 *via* the NACHR-YAP1-E2F1 signaling axis that maintains the characteristic of NSCLC tumor stem cells (Schaal et al., 2018).

Moreover, tobacco can induce more incidence of SNV, including *TP53*, *TTN*, *MUC16*, *SYNE1*, *CSMD13*, *RYR2*, *USH2A,* and *FLG*, which may play a pivotal role in mediating cell malignancy and tumor progression that cannot be reversed even after quitting smoking in a certain gene. We noted that non-smokers were substantially more sensitive to multiple chemotherapeutic drugs than smokers. Similarly, previous studies have found that smoking can induce mutations in multiple genes, which in turn can induce patients to become resistant to chemotherapy drugs (Alexandrov et al., 2016; Shang et al., 2020). For instance, *TP53*-mutations type can interact with *BCAR1* to promote tumor cell invasion, leading to poor prognosis (Guo et al., 2020). In comparison, the incidence of *TP53* mutation was higher in smokers than in non-smokers. It was also observed that the frequency of *TP53* mutation increased with the increase of smoking amount (Halvorsen et al., 2016). A study by Yu et al. (2019) analyzed somatic mutations in 100 cases of NSCLC, whose results revealed that a variety of gene mutations such as *TTN*, *CSMD3*, *RYR2*, *USH2A*, and *ZFHX4* were different in patients with different smoking histories, and thus the mutation incidence was higher in smokers than that in non-smokers. Likewise, Shang et al. found that the mutation frequency of *CDKN2A*, *FAT1*, *FGFR1*, *NFE2L1*, *CCNE1*, *CCND1*, *SMARCA4*, *KEAP1*, *KMT2C*, and *STK11* was higher in smokers compared with non-smokers (Shang et al., 2020). According to the literature, chromosome instability can lead to CNV and genetic heterogeneity, which may trigger the occurrence of cancer (Myllykangas et al., 2008). In this study, we unearthed that tobacco causes a higher incidence of CNV, mainly occurring on chromosomes 3, 8, 1, 5, 9, 19, and 4. The gain of the 3q26 locus was remarkably related to the occurrence of human squamous cell carcinoma, including LUSC and HNSC. Hence, the gain of 3q26 was significantly associated with smoking (Li et al., 2020a). *CDKN2A* (9p21.3) encodes P16 protein that is a tumor suppressor. Studies have shown that *CDKN2A* loss and abnormal expression of P16 are associated with the occurrence of various malignant tumors (Kettunen et al., 2019). Smoking-related HNSC tumors indicated a large number of *CDKN2A* losses, suggesting that smoking may induce *CDKN2A* CNV loss (Cancer Genome Atlas, 2015). In oral cancer, *TP63* CNV was significantly associated with smoking history, while the incidence of *TP63* CNV gain was considerably increased among smokers (Pattle et al., 2017). A recent study by Tom et al. reported that loss of 19q13.42 occurred at a significantly higher rate in recurrent anal cancer than in primary tumors, implying that a loss of 19q13.42 may promote tumor recurrence (Cacheux et al., 2018). The loss of chromosome segment 8p23.3 is markedly associated with the development and progression ofBLCA, resulting in poor tumor staging (Muscheck et al., 2000). In another study, Joseph et al. (2020) found that loss of *ARHGEF10* was found in more than 30% of pancreatic cancers (PC), whose loss led to enhanced subcutaneous tumor growth in the mouse model as well as increased proliferation, invasion, and motility of PC cell lines *in vitro*, and also enhanced tumor metastatic spread in the mouse model. The gain of *PLD1*, which is prevalent in LUSC, is considered as a new biomarker for LUSC (Mendez and Ramirez, 2013).

Smoking is associated with the induction of chemical resistance in different types of cancers, namely, CRC (Lee et al., 2016), HSNC (Shen et al., 2010), PC (Lee et al., 2016), and BLCA (Chen et al., 2010). In particular, smoking promotes chemotherapy-resistant and anti-apoptotic effects on breast cancer cells by signaling cascades of STAT3, galectin-3, and nicotine acetylcholine receptors (Guha et al., 2014). Tobacco may also alter the pharmacodynamics of anti-cancer drugs (Willey et al., 1997; Villard et al., 1998). For example, in lung cancer, smokers who were treated with chemotherapeutic drugs showed more rapid elimination than non-smokers, requiring an increase in dose to achieve the same therapeutic effect (O’Malley et al., 2014). Nicotine has been established to promote XIAP protein stabilization and surviving transcriptional induction through the Akt pathway, thereby inhibiting the apoptosis effects of chemotherapeutic drugs on NSCLC tumor cells (Dasgupta et al., 2006). The components pyrazine, 2-ethylpyridine, and 3-ethylpyridine in tobacco can induce multi-drug resistance in LUAD tumor cells. The induction is enhanced in hypoxia (Liu et al., 2015). In BLCA, smoking induces tumor growth and mTOR inhibitor resistance through activation of the PI3K/Akt/mTOR signaling pathway (Yuge et al., 2015). Jin et al. (2004) demonstrated that nicotine-induced multisite phosphorylation of BAD may be the cause of resistance to PKC and MEK inhibitors in human lung cancer.

Nicotine, the major component of cigarette smoke, can stimulate the expression of VEGF in endothelial cells, thereby promoting endothelial cell proliferation, migration, and angiogenesis (Zhang et al., 2015). In addition, smoking can activate the combination of VEGF promoter and MZF1 to induce VEGF expression (Krüger et al., 2020). Yuge et al. pointed that smoking activates the PI3K/Akt/mTOR signaling pathway in BLCA to promote tumor cell growth and develop resistance to chemotherapeutic drugs (Yuge et al., 2015).

In addition, we identified several core genes that may contribute to tobacco-related tumors by constructing a ceRNA network. Mir-301b targets *FOXF2*, *PTEN*, and *COL2A1*, regulating the proliferation, colony formation, cell migration, invasion, chemotherapy resistance, and tumor growth of ductal invasive breast cancer cells without lymph node metastasis (Shi et al., 2011). Mir-301b is found to be upregulated in certain cancers, such as PC, lung cancer, oral cancer, and liver cancer (Miko et al., 2009; Lu et al., 2012; Wang et al., 2016). For ESCA, mir-205-5p was largely higher in smokers (Stánitz et al., 2015). For chronic lung disease, the mir-132-3p was significantly upregulated in smokers, thus inducing increased concentrations of inflammatory cytokines (interleukin-1β and tumor necrosis factor-α) (Diao et al., 2018). Furthermore, cigarette tobacco extract can induce the expression of mir-132-3p (Singh et al., 2020). Numerous studies have highlighted that mir-1271-5p and mir-137 may be associated with tumor proliferation, infiltration, and drug resistance, which may form a new target of cancer therapy (Liu et al., 2017; Lu et al., 2017; Jiang et al., 2018; Qiu et al., 2019; Wang et al., 2019; Wu et al., 2019; Dang et al., 2020; Li et al., 2020b).

Epigenetic disorders are associated with the occurrence and progression of cancer. In gastric cancer, levels of *GBP6* methylation are negatively correlated with their mRNA expression. Consequently, patients with high levels of *GBP6* methylation are significantly associated with poor prognoses (Peng et al., 2020). Also, *GBP6* is associated with oral cancer and HNSC therefore can be used as a prognostic marker (Liu et al., 2020; Wu et al., 2020). *ITGA5* has been reported to mediate the initial adhesion process in ovarian and CRC (Ohyagi-Hara et al., 2013; Yoo et al., 2016). In breast cancer, the increased levels of *ITAG5* promoter methylation led to the decrease of *ITAG5* expression, thus inhibiting the growth, development, and cell migration of breast cancer cells (Fang et al., 2010). However, a high *ITGA5* expression was associated with malignant characteristics of BLCA and HNSC (Deng et al., 2019; Yan and Ye, 2019). In oral cancer, it has been found that *NR2F2* is hypermethylated in cancer tissue compared to normal tissue, which may play a critical role in oral cancer. In glioma, *NR2F2* hypermethylation is a differentially expressed methylated gene between glioma patients with better prognosis and poor prognosis, which is enriched in diseases and disorders in both molecular and cellular aspects (Shinawi et al., 2013). Additionally, *PPP1R18* encodes a defense protein that is tightly localized to cytoskeleton proteins. Significant reductions in *PPP1R18* methylation have been recorded in patients with severe liver fibrosis, suggesting that epigenetic disorders are involved in the progression of the disease (Zeybel et al., 2016). *SLC6A15* methylation level was significantly higher in CRC, although there was no significant correlation between the *SLC6A15* methylation level and its mRNA expression level (Kim et al., 2011; Mitchell et al., 2014). Meanwhile, the *SLC6A15* mRNA expression was significantly reduced in ovarian cancer cell lines with a chemotherapy-resistant phenotype. Up to now, there are few reports on the association between *EIF5A2*, *HGD*, *HS6ST1*, *PLS1*, *PTHLH,* and *YEATS2* methylation modification and tumor. The overexpression of *EIF5A2* was associated with invasion, metastasis, and other malignant phenotypes of various cancers, suggesting that *EIF5A2* may be a potential therapeutic target (Chen et al., 2018; Ba et al., 2019; Dong et al., 2019). In addition, *EIF5A2* regulates the resistance of gastric cancer cells to cisplatin by mediating epithelial–stromal transformation (Sun et al., 2018). The downregulation of *HGD* expression was found to be associated with less metastasis, as well as better prognosis, pathological grade, and clinical stage of cholangiocarcinoma patients (Aukkanimart et al., 2015). The *PLS1* was overexpressed in CRC patients and associated with lymph node metastasis and a poor prognosis. The *PLS1* can also induce the migration and invasion of CRC cells as well as the metastasis to the liver and lung. In addition, the *PLS1* also enhanced the expression of matrix metalloproteinases 9 and 2, which were key factors in CRC metastasis (Zhang et al., 2020). Compared with the normal tissue, *PTHLH* was significantly overexpressed in HNSC and was associated with poor prognosis in HNSC. Meanwhile, the increased expression of the *PTHLH* can induce the cell cycle progression of tumor cells and actively regulate the expression of core proteins (Chang et al., 2017). The increased expression of *HS6ST1* mRNA during the progression of cartilage tumors suggests that *HS6ST1* may promote the formation of malignant phenotypes of cartilage tumors (Waaijer et al., 2012). The inhibition of *YEATS2* mRNA expression can reduce the proliferation and migration of PC cells. Meanwhile, the hypoxia-inducible factor 1α (HIF1α) regulates the expression of *YEATS2* mRNA by binding to the hypoxia response element of *YEATS2*. HIF1α was co-expressed with *YEATS2* in PC. In turn, overexpressed *YEATS2* can block the inhibitory effect of HIF1α silencing on PC cell proliferation and migration under hypoxia (Zeng et al., 2021).

Next, we developed a prognostic model for tumor patients. Our findings elucidated that the OS of patients in the high-risk score group was poor compared with that of patients in the low-risk score group. It is to be noted, the high-risk score was an independent risk factor for OS. The results of ROC showed that the model has good ability to predict OS. We further constructed a nomogram for each cancer type, including the prognostic model and clinical features, to predict OS. Both calibration diagrams and C-indexes confirmed that the nomograms were reliable and highly accurate. Moreover, the prognostic model has a wide clinical applicability for predicting the DSS and PFI of cancer patients. We also drew the nomograms and verified that they had good ability in forecasting the DSS and PFI of patients.

Nevertheless, despite these intriguing results, this study has some shortcomings. First, we did not analyze the influence of smoking time and the number of cigarettes on the study. Second, we did not evaluate the influence of different durations of quitting smoking on the study. Finally, we did not validate the results *in vivo* and vitro experiments.

In summary, we systematically studied the molecular level differences among non-smokers, former smokers, and current smokers. We found that smoking cessation can reduce the risk of poor prognosis in patients. However, at the same time, tobacco induces SNVs and CNVs, which are changes that can be reversed by smoking cessation. Furthermore, smoking can activate the immune function of patients, while continuous smoking may induce a decline in immune indicators. Therefore, based on this, we recommend that further functional experiments are needed to verify our findings.

## Data Availability

The original contributions presented in the study are included in the article/Supplementary Material; further inquiries can be directed to the corresponding author.

## Supplementary Material

Supplementary Figure 1
* p*-Value and χ^2^ of top 30 genes incidence of **(A)** SNV, **(B)** CNV gain and **(C)** CNV loss among patients with different smoking histories.

Supplementary Figure 2 KM curves showed a difference in OS among patients with different risk scores, and an independent prognostic analysis was applied to analyze the effects of risk scores on OS along with other clinical features.

Supplementary Figure 3 ROC curves of the ability of the smoking-related model and other clinical features to predict patient’s 1-, 3- and 5-years OS in each cancer type.

Supplementary Figure 4 Construction of nomograms for predicting OS in patients and evaluation of its accuracy. Nomograms were developed by combining the smoking-related model with age, gender, and stage to predict the 1-, 3- and 5-year OS of patients in each cancer type. Calibration diagrams were employed to establish the consistency of the nomogram prediction OS of patients with real results.

Supplementary Figure 5 KM curves showed a difference in DSS among patients with different risk scores, and an independent prognostic analysis was applied to analyze the effects of risk scores on DSS along with other clinical features.

Supplementary Figure 6 KM curves showed a difference in PFI among patients with different risk scores, and an independent prognostic analysis was applied to analyze the effects of risk scores on PFI along with other clinical features.

Supplementary Figure 7 ROC curves of the predictive ability of the prognostic model predicting 1-, 3- and 5-years DSS **(A–G)** and PFI **(H–N)** of patients in each cancer type.

Supplementary Figure 8 Construction of nomograms for predicting DSS of each cancer patient as well as evaluation of its accuracy. Nomograms were constructed by use of the smoking-related model and other clinical indicators to predict the DSS of each cancer patient. Calibration curves were used to evaluate the consistency of nomogram prediction patient DSS with real results.

Supplementary Figure 9 Construction of nomograms for predicting PFI of each cancer patient as well as evaluation of its accuracy. Nomograms were constructed by use of the smoking-related model and other clinical indicators to predict the PFI of each cancer patient. Calibration curves were used to evaluate the consistency of nomogram prediction patient PFI with real results.

Supplementary Table 1 Information about the included samples.

Supplementary Table 2 The results of GSEA between patients with any two smoking histories (**Sheet 1**). Identification and Intersection of differentially expressed genes between any two smoking histories in patients with various smoking histories (lncRNA & mRNA: **Sheet 2**, miRNA: **Sheet 3**, methylated genes: **Sheet 4**). Genes associated with OS were obtained through cox regression analysis (**Sheet 5**). Risk scores for all samples were obtained via the prognostic model (**Sheet 6**).

## Glossary

AUC: area under the curve
BCR: B-cell receptor
BLCA: bladder urothelial carcinoma
CCR: chemokine receptor
CeRNA: competing endogenous RNA
CESC: cervical squamous cell carcinoma and endocervical adenocarcinoma
C-index: concordance index
CNVs: copy number variations
Cor: correlation coefficient
CRC: colorectal cancer
CTA: cancer testis antigen
DCs: dendritic cells
DElncRNAs: differentially expressed lncRNAs
DEmiRNAs: differentially expressed miRNAs
DEmRNAs: differentially expressed mRNAs
DMPsi: differentially methylated probes-based stemness index
DSS: disease-specific survival
ENHsi: enhancer-based stemness index
EREG-mDNAsi: DNA methylation-based epigenetically regulated-mDNAsi
EREG-mRNAsi: RNA expression-based epigenetically regulated-mRNAsi
ESCA: esophageal carcinoma
FDR *q*-value: false discovery rate *q*-value
GDSC: genomics of drug sensitivity in cancer
GISTIC: genomic identification of significant targets in cancer
GSEA: gene set enrichment analysis
HNSC: head and neck squamous cell carcinoma
HR: hazard ratio
HRD: homologous recombination defects
HIF1α: hypoxia-inducible factor 1α
IC50: half maximal inhibitory concentration
IFN: interferon
KIRP: kidney renal papillary cell carcinoma
LUAD: lung adenocarcinoma
LUSC: lung squamous cell carcinoma
mDNAsi: DNA methylation stemness indices
mRNAsi: mRNA stemness indices
NES: normalized enrichment score
NK: natural killer
NOM *p*-value: nominal *p*-value
OS: overall survival
PC: pancreatic cancer
PFI: progression-free interval
ROC: receiver operating characteristic
SNVs: simple nucleotide variations
ssGSEA: single-sample gene set enrichment analysis
TCGA: the cancer genome atlas
TMB: tumor mutation burden

## References

[B1] Ahmad S. Zafar I. Mariappan N. Husain M. Wei C.-C Vetal N. (2019). Acute Pulmonary Effects of Aerosolized Nicotine. Am. J. Physiology-Lung Cell Mol. Physiol. 316, L94–L104. 10.1152/ajplung.00564.2017 PMC6383503 30358437

[B2] Alexandrov L. B. Ju Y. S. Haase K. Van Loo P. Martincorena I. Nik-Zainal S. (2016). Mutational Signatures Associated with Tobacco Smoking in Human Cancer. Science 354, 618–622. 10.1126/science.aag0299 27811275 PMC6141049

[B3] An Y. Kiang A. Lopez J. P. Kuo S. Z. Yu M. A. Abhold E. L. (2012). Cigarette Smoke Promotes Drug Resistance and Expansion of Cancer Stem Cell-like Side Population. PLoS One 7, e47919. 10.1371/journal.pone.0047919 23144836 PMC3489897

[B4] Arnson Y. Shoenfeld Y. Amital H. (2010). Effects of Tobacco Smoke on Immunity, Inflammation and Autoimmunity. J. Autoimmun. 34, J258–J265. 10.1016/j.jaut.2009.12.003 20042314

[B5] Aukkanimart R. Boonmars T. Juasook A. Sriraj P. Boonjaraspinyo S. Wu Z. (2015). Altered Expression of Oxidative Metabolism Related Genes in Cholangiocarcinomas. Asian Pac. J. Cancer Prev. 16, 5875–5881. 10.7314/apjcp.2015.16.14.5875 26320466

[B6] Ba M.-C. Ba Z. Cui S.-Z. Gong Y.-F. Chen C. Lin K.-P. (2019). Thermo-chemotherapy Inhibits the Proliferation and Metastasis of Gastric Cancer Cells via Suppression of EIF5A2 Expression. Ott 12, 6275–6284. 10.2147/OTT.S215590 PMC6691964 31496731

[B7] Barbie D. A. Tamayo P. Boehm J. S. Kim S. Y. Moody S. E. Dunn I. F. (2009). Systematic RNA Interference Reveals that Oncogenic KRAS-Driven Cancers Require TBK1. Nature 462, 108–112. 10.1038/nature08460 19847166 PMC2783335

[B8] Bozinovski S. Seow H. J. Chan S. P. J. Anthony D. McQualter J. Hansen M. (2015). Innate Cellular Sources of interleukin-17A Regulate Macrophage Accumulation in Cigarette- Smoke-Induced Lung Inflammation in Mice. Clin. Sci. (Lond) 129, 785–796. 10.1042/CS20140703 26201093 PMC4613531

[B9] Cacheux W. Tsantoulis P. Briaux A. Vacher S. Mariani P. Richard‐Molard M. (2018). Array Comparative Genomic Hybridization Identifies High Level of PI3K/Akt/mTOR Pathway Alterations in Anal Cancer Recurrences. Cancer Med. 7, 3213–3225. 10.1002/cam4.1533 PMC6051172 29804324

[B10] Cancer Genome Atlas N. (2015). Comprehensive Genomic Characterization of Head and Neck Squamous Cell Carcinomas. Nature 517, 576–582. 10.1038/nature14129 25631445 PMC4311405

[B11] Chang W.-M. Lin Y.-F. Su C.-Y. Peng H.-Y. Chang Y.-C. Hsiao J.-R. (2017). Parathyroid Hormone-like Hormone Is a Poor Prognosis Marker of Head and Neck Cancer and Promotes Cell Growth via RUNX2 Regulation. Sci. Rep. 7, 41131. 10.1038/srep41131 28120940 PMC5264159

[B12] Chen C. Zhang B. Wu S. Song Y. Li J. (2018). Knockdown of EIF5A2 Inhibits the Malignant Potential of Non-small Cell Lung Cancer Cells. Oncol. Lett. 15, 4541–4549. 10.3892/ol.2018.7832 29541224 PMC5835861

[B13] Chen R.-J. Ho Y.-S. Guo H.-R. Wang Y.-J. (2010). Long-term Nicotine Exposure-Induced Chemoresistance Is Mediated by Activation of Stat3 and Downregulation of ERK1/2 via nAChR and Beta-Adrenoceptors in Human Bladder Cancer Cells. Toxicol. Sci. 115, 118–130. 10.1093/toxsci/kfq028 20106947

[B14] Cui W.-Y. Li M. D. (2010). Nicotinic Modulation of Innate Immune Pathways via α7 Nicotinic Acetylcholine Receptor. J. Neuroimmune Pharmacol. 5, 479–488. 10.1007/s11481-010-9210-2 20387124

[B15] Dang Y. Liu T. Yan J. Reinhardt J. D. Yin C. Ye F. (2020). Gastric Cancer Proliferation and Invasion Is Reduced by Macrocalyxin C via Activation of the miR-212-3p/Sox6 Pathway. Cell Signal. 66, 109430. 10.1016/j.cellsig.2019.109430 31726103

[B16] Dasgupta P. Kinkade R. Joshi B. Decook C. Haura E. Chellappan S. (2006). Nicotine Inhibits Apoptosis Induced by Chemotherapeutic Drugs by Up-Regulating XIAP and Survivin. Proc. Natl. Acad. Sci. 103, 6332–6337. 10.1073/pnas.0509313103 16601104 PMC1458878

[B17] Deng Y. Wan Q. Yan W. (2019). Integrin α5/ITGA5 Promotes the Proliferation, Migration, Invasion and Progression of Oral Squamous Carcinoma by Epithelial-Mesenchymal Transition. Cmar 11, 9609–9620. 10.2147/CMAR.S223201 PMC6859091 32009816

[B18] Diao X. Zhou J. Wang S. Ma X. (2018). Upregulation of miR-132 Contributes to the Pathophysiology of COPD via Targeting SOCS5. Exp. Mol. Pathol. 105, 285–292. 10.1016/j.yexmp.2018.10.002 30292646

[B19] Doll R. Peto R. Boreham J. Sutherland I. (2004). Mortality in Relation to Smoking: 50 years' Observations on Male British Doctors. BMJ 328, 1519. 10.1136/bmj.38142.554479.ae 15213107 PMC437139

[B20] Dong J. S. Wu B. Zha Z. L. (2019). MicroRNA-588 Regulates Migration Capacity and Invasiveness of Renal Cancer Cells by Targeting EIF5A2. Eur. Rev. Med. Pharmacol. Sci. 23, 10248–10256. 10.26355/eurrev_201912_19662 31841179

[B21] Dugué P.-A. Hodge A. M. Wong E. M. Joo J. E. Jung C.-H. Hopper J. L. (2020). Methylation marks of Prenatal Exposure to Maternal Smoking and Risk of Cancer in Adulthood. Int. J. Epidemiol. 50, 105–115. 10.1093/ije/dyaa210 33169152

[B22] Eriksen M. P. LeMaistre C. A. Newell G. R. (1988). Health Hazards of Passive Smoking. Annu. Rev. Public Health. 9, 47–70. 10.1146/annurev.pu.09.050188.000403 3288240

[B23] Fang Z. Yao W. Xiong Y. Zhang J. Liu L. Li J. (2010). Functional Elucidation and Methylation-Mediated Downregulation of ITGA5 Gene in Breast Cancer Cell Line MDA-MB-468. J. Cell. Biochem. 110, 1130–1141. 10.1002/jcb.22626 20564209

[B24] Foerster B. Pozo C. Abufaraj M. Mari A. Kimura S. D’Andrea D. (2018). Association of Smoking Status with Recurrence, Metastasis, and Mortality Among Patients with Localized Prostate Cancer Undergoing Prostatectomy or Radiotherapy. JAMA Oncol. 4, 953–961. 10.1001/jamaoncol.2018.1071 29800115 PMC6145736

[B25] Friedman J. Hastie T. Tibshirani R. (2010). Regularization Paths for Generalized Linear Models via Coordinate Descent. J. Stat. Softw. 33, 1–22. 10.18637/jss.v033.i01 20808728 PMC2929880

[B26] GBDRF Collaborators. Global burden of 87 Risk Factors in 204 Countries and Territories, 1990-2019: a Systematic Analysis for the Global Burden of Disease Study 2019. Lancet. (2020) 396:1223–1249. 10.1016/S0140-6736(20)30752-2 33069327 PMC7566194

[B27] Geeleher P. Cox N. J. Huang R. (2014). Clinical Drug Response Can Be Predicted Using Baseline Gene Expression Levels and *In Vitro* Drug Sensitivity in Cell Lines. Genome Biol. 15, R47. 10.1186/gb-2014-15-3-r47 24580837 PMC4054092

[B28] Guha P. Bandyopadhyaya G. Polumuri S. K. Chumsri S. Gade P. Kalvakolanu D. V. (2014). Nicotine Promotes Apoptosis Resistance of Breast Cancer Cells and Enrichment of Side Population Cells with Cancer Stem Cell-like Properties via a Signaling cascade Involving Galectin-3, α9 Nicotinic Acetylcholine Receptor and STAT3. Breast Cancer Res. Treat. 145, 5–22. 10.1007/s10549-014-2912-z 24668500 PMC4028025

[B29] Guo A. K. Itahana Y. Seshachalam V. P. Chow H. Y. Ghosh S. Itahana K. (2020). Mutant TP53 Interacts with BCAR1 to Contribute to Cancer Cell Invasion. Br. J. Cancer. 124, 299–312. 10.1038/s41416-020-01124-9 33144694 PMC7782524

[B30] Halvorsen A. R. Silwal-Pandit L. Meza-Zepeda L. A. Vodak D. Vu P. Sagerup C. (2016). TP53 Mutation Spectrum in Smokers and Never Smoking Lung Cancer Patients. Front. Genet. 07, 85. 10.3389/fgene.2016.00085 PMC4863128 27242894

[B31] Heagerty P. J. Lumley T. Pepe M. S. (2000). Time-dependent ROC Curves for Censored Survival Data and a Diagnostic Marker. Biometrics 56, 337–344. 10.1111/j.0006-341x.2000.00337.x 10877287

[B32] Husgafvel-Pursiainen K. Boffetta P. Kannio A. Nyberg F. Pershagen G. Mukeria A. (2000). p53 Mutations and Exposure to Environmental Tobacco Smoke in a Multicenter Study on Lung Cancer. Cancer Res. 60, 2906–2911. 10850436

[B33] Inamura K. Yokouchi Y. Kobayashi M. Sakakibara R. Ninomiya H. Subat S. (2017). Tumor B7-H3 (CD276) Expression and Smoking History in Relation to Lung Adenocarcinoma Prognosis. Lung Cancer. 103, 44–51. 10.1016/j.lungcan.2016.11.013 28024695

[B34] Jiang H. Zhang H. Hu X. Li W. (2018). Knockdown of Long Non-coding RNA XIST Inhibits Cell Viability and Invasion by Regulating miR-137/PXN axis in Non-small Cell Lung Cancer. Int. J. Biol. Macromolecules 111, 623–631. 10.1016/j.ijbiomac.2018.01.022 29337100

[B35] Jin Z. Gao F. Flagg T. Deng X. (2004). Nicotine Induces Multi-Site Phosphorylation of Bad in Association with Suppression of Apoptosis. J. Biol. Chem. 279, 23837–23844. 10.1074/jbc.M402566200 15037618

[B36] Joseph J. Radulovich N. Wang T. Raghavan V. Zhu C.-Q. Tsao M.-S. (2020). Rho Guanine Nucleotide Exchange Factor ARHGEF10 Is a Putative Tumor Suppressor in Pancreatic Ductal Adenocarcinoma. Oncogene 39, 308–321. 10.1038/s41388-019-0985-1 31477830

[B37] Kenfield S. A. Stampfer M. J. Chan J. M. Giovannucci E. (2011). Smoking and Prostate Cancer Survival and Recurrence. JAMA 305, 2548–2555. 10.1001/jama.2011.879 21693743 PMC3562349

[B38] Kettunen E. Savukoski S. Salmenkivi K. Böhling T. Vanhala E. Kuosma E. (2019). CDKN2A Copy Number and P16 Expression in Malignant Pleural Mesothelioma in Relation to Asbestos Exposure. BMC Cancer 19, 507. 10.1186/s12885-019-5652-y 31138176 PMC6537412

[B39] Kim Y.-H. Lee H. C. Kim S.-Y. Yeom Y. I. Ryu K. J. Min B.-H. (2011). Epigenomic Analysis of Aberrantly Methylated Genes in Colorectal Cancer Identifies Genes Commonly Affected by Epigenetic Alterations. Ann. Surg. Oncol. 18, 2338–2347. 10.1245/s10434-011-1573-y 21298349 PMC3393129

[B40] Krüger M. Metzger C. Al‐Nawas B. Kämmerer P. W. Brieger J. (2020). Cigarette Smoke Modulates Binding of the Transcription Factor MZF1 to the VEGF Promoter and Regulates VEGF Expression in Dependence of Genetic Variation SNP 405. J. Oral Pathol. Med. 49, 780–786. 10.1111/jop.13038 32449233

[B41] Kulhánová I. Forman D. Vignat J. Espina C. Brenner H. Storm H. H. (2020). Tobacco-related Cancers in Europe: The Scale of the Epidemic in 2018. Eur. J. Cancer. 139, 27–36. 10.1016/j.ejca.2020.07.024 32957011

[B42] Lee T.-Y. Liu C.-L. Chang Y.-C. Nieh S. Lin Y.-S. Jao S.-W. (2016). Increased Chemoresistance via Snail-Raf Kinase Inhibitor Protein Signaling in Colorectal Cancer in Response to a Nicotine Derivative. Oncotarget 7, 23512–23520. 10.18632/oncotarget.8049 26992205 PMC5029643

[B43] Li D. Wang X. Lu S. Wang P. Wang X. Yin W. (2020). Integrated Analysis Revealing Genome-wide C-hromosomal C-opy N-umber V-ariation in S-upraglottic L-aryngeal S-quamous C-ell C-arcinoma. Oncol. Lett. 20, 1201–1212. 10.3892/ol.2020.11653 32724360 PMC7377034

[B44] Li X. Li J. Wu P. Zhou L. Lu B. Ying K. (2018). Smoker and Non-smoker Lung Adenocarcinoma Is Characterized by Distinct Tumor Immune Microenvironments. Oncoimmunology 7, e1494677. 10.1080/2162402X.2018.1494677 30288364 PMC6169585

[B45] Li X. Zou Z.-Z. Wen M. Xie Y.-Z. Peng K.-J. Luo T. (2020). ZLM-7 Inhibits the Occurrence and Angiogenesis of Breast Cancer through miR-212-3p/Sp1/VEGFA Signal axis. Mol. Med. 26, 109. 10.1186/s10020-020-00239-2 33187481 PMC7666510

[B46] Liu M. Poo W.-K. Lin Y.-l. (2015). Pyrazine, 2-ethylpyridine, and 3-ethylpyridine Are Cigarette Smoke Components that Alter the Growth of normal and Malignant Human Lung Cells, and Play a Role in Multidrug Resistance Development. Exp. Mol. Pathol. 98, 18–26. 10.1016/j.yexmp.2014.11.008 25449333

[B47] Liu P.-F. Chen H.-C. Shu C.-W. Sie H.-C. Lee C.-H. Liou H.-H. (2020). Guanylate-binding Protein 6 Is a Novel Biomarker for Tumorigenesis and Prognosis in Tongue Squamous Cell Carcinoma. Clin. Oral Invest. 24, 2673–2682. 10.1007/s00784-019-03129-y 31707626

[B48] Liu X. Chen L. Tian X. D. Zhang T. (2017). MiR-137 and its Target TGFA Modulate Cell Growth and Tumorigenesis of Non-small Cell Lung Cancer. Eur. Rev. Med. Pharmacol. Sci. 21, 511–517. 28239819

[B49] Lu A.-Q. Lv B. Qiu F. Wang X.-Y. Cao X.-H. (2017). Upregulation of miR-137 Reverses Sorafenib Resistance and Cancer-Initiating Cell Phenotypes by Degrading ANT2 in Hepatocellular Carcinoma. Oncol. Rep. 37, 2071–2078. 10.3892/or.2017.5498 28350139

[B50] Lu Y.-C. Chen Y.-J. Wang H.-M. Tsai C.-Y. Chen W.-H. Huang Y.-C. (2012). Oncogenic Function and Early Detection Potential of miRNA-10b in Oral Cancer as Identified by microRNA Profiling. Cancer Prev. Res. 5, 665–674. 10.1158/1940-6207.CAPR-11-0358 22318752

[B51] Maeno T. Houghton A. M. Quintero P. A. Grumelli S. Owen C. A. Shapiro S. D. (2007). CD8+ T Cells Are Required for Inflammation and Destruction in Cigarette Smoke-Induced Emphysema in Mice. J. Immunol. 178, 8090–8096. 10.4049/jimmunol.178.12.8090 17548647

[B52] Malta T. M. Sokolov A. Gentles A. J. Burzykowski T. Poisson L. Weinstein J. N. (2018). Machine Learning Identifies Stemness Features Associated with Oncogenic Dedifferentiation. Cell 173, 338–e15. 10.1016/j.cell.2018.03.034 29625051 PMC5902191

[B53] Manjer J. Andersson I. Berglund G. Bondesson L. Garne J. P. Janzon L. (2000). Survival of Women with Breast Cancer in Relation to Smoking. Eur. J. Surg. 166, 852–858. 10.1080/110241500447227 11097150

[B54] Mayakonda A. Lin D.-C. Assenov Y. Plass C. Koeffler H. P. (2018). Maftools: Efficient and Comprehensive Analysis of Somatic Variants in Cancer. Genome Res. 28, 1747–1756. 10.1101/gr.239244.118 30341162 PMC6211645

[B55] Mendez P. Ramirez J. L. (2013). Copy Number Gains of FGFR1 and 3q Chromosome in Squamous Cell Carcinoma of the Lung. Transl Lung Cancer Res. 2, 101–111. 10.3978/j.issn.2218-6751.2013.03.05 25806221 PMC4369856

[B56] Mermel C. H. Schumacher S. E. Hill B. Meyerson M. L. Beroukhim R. Getz G. (2011). GISTIC2.0 Facilitates Sensitive and Confident Localization of the Targets of Focal Somatic Copy-Number Alteration in Human Cancers. Genome Biol. 12, R41. 10.1186/gb-2011-12-4-r41 21527027 PMC3218867

[B57] Miko E. Czimmerer Z. Csánky E. Boros G. Buslig J. Dezső B. (2009). Differentially Expressed microRNAs in Small Cell Lung Cancer. Exp. Lung Res. 35, 646–664. 10.3109/01902140902822312 19895320

[B58] Mitchell S. M. Ross J. P. Drew H. R. Ho T. Brown G. S. Saunders N. F. (2014). A Panel of Genes Methylated with High Frequency in Colorectal Cancer. BMC Cancer 14, 54. 10.1186/1471-2407-14-54 24485021 PMC3924905

[B59] Moreira D. M. Nickel J. C. Gerber L. Muller R. L. Andriole G. L. Castro-Santamaria R. (2015). Smoking Is Associated with Acute and Chronic Prostatic Inflammation: Results from the REDUCE Study. Cancer Prev. Res. 8, 312–317. 10.1158/1940-6207.CAPR-14-0260 25644151

[B60] Motz G. T. Eppert B. L. Wortham B. W. Amos-Kroohs R. M. Flury J. L. Wesselkamper S. C. (2010). Chronic Cigarette Smoke Exposure Primes NK Cell Activation in a Mouse Model of Chronic Obstructive Pulmonary Disease. J.I. 184, 4460–4469. 10.4049/jimmunol.0903654 20228194

[B61] Muscheck M. Sükösd F. Pesti T. Kovacs G. (2000). High Density Deletion Mapping of Bladder Cancer Localizes the Putative Tumor Suppressor Gene between Loci D8S504 and D8S264 at Chromosome 8p23.3. Lab. Invest 80, 1089–1093. 10.1038/labinvest.3780114 10908154

[B62] Myllykangas S. Tikka J. Böhling T. Knuutila S. Hollmén J. (2008). Classification of Human Cancers Based on DNA Copy Number Amplification Modeling. BMC Med. Genomics 1, 15. 10.1186/1755-8794-1-15 18477412 PMC2397431

[B63] Nadigel J. Préfontaine D. Baglole C. J. Maltais F. Bourbeau J. Eidelman D. H. (2011). Cigarette Smoke Increases TLR4 and TLR9 Expression and Induces Cytokine Production from CD8+T Cells in Chronic Obstructive Pulmonary Disease. Respir. Res. 12, 149. 10.1186/1465-9921-12-149 22070100 PMC3260101

[B64] Ohyagi-Hara C. Sawada K. Kamiura S. Tomita Y. Isobe A. Hashimoto K. (2013). miR-92a Inhibits Peritoneal Dissemination of Ovarian Cancer Cells by Inhibiting Integrin α5 Expression. Am. J. Pathol. 182, 1876–1889. 10.1016/j.ajpath.2013.01.039 23499550

[B65] O’Malley M. King A. N. Conte M. Ellingrod V. L. Ramnath N. (2014). Effects of Cigarette Smoking on Metabolism and Effectiveness of Systemic Therapy for Lung Cancer. J. Thorac. Oncol. 9, 917–926. 10.1097/JTO.0000000000000191 24926542

[B66] Ordóñez-Mena J. M. Walter V. Schöttker B. Jenab M. O’Doherty M. G. Kee F. (2018). Impact of Prediagnostic Smoking and Smoking Cessation on Colorectal Cancer Prognosis: a Meta-Analysis of Individual Patient Data from Cohorts within the CHANCES Consortium. Ann. Oncol. 29, 472–483. 10.1093/annonc/mdx761 29244072 PMC6075220

[B67] Parsons A. Daley A. Begh R. Aveyard P. (2010). Influence of Smoking Cessation after Diagnosis of Early Stage Lung Cancer on Prognosis: Systematic Review of Observational Studies with Meta-Analysis. BMJ 340, b5569. 10.1136/bmj.b5569 20093278 PMC2809841

[B68] Pattle S. B. Utjesanovic N. Togo A. Wells L. Conn B. Monaghan H. (2017). Copy Number Gain of 11q13.3 Genes Associates with Pathological Stage in Hypopharyngeal Squamous Cell Carcinoma. Genes Chromosomes Cancer 56, 185–198. 10.1002/gcc.22425 27750372

[B69] Peng Y. Wu Q. Wang L. Wang H. Yin F. (2020). A DNA Methylation Signature to Improve Survival Prediction of Gastric Cancer. Clin. Epigenet. 12, 15. 10.1186/s13148-020-0807-x PMC6972030 31959204

[B70] Prueitt R. L. Wallace T. A. Glynn S. A. Yi M. Tang W. Luo J. (2016). An Immune-Inflammation Gene Expression Signature in Prostate Tumors of Smokers. Cancer Res. 76, 1055–1065. 10.1158/0008-5472.CAN-14-3630 26719530 PMC4775384

[B71] Qian W. Kong X. Zhang T. Wang D. Song J. Li Y. (2018). Cigarette Smoke Stimulates the Stemness of Renal Cancer Stem Cells via Sonic Hedgehog Pathway. Oncogenesis 7, 24. 10.1038/s41389-018-0029-7 29540668 PMC5852977

[B72] Qiu H. B. Yang K. Yu H. Y. Liu M. (2019). Downregulation of Long Non-coding RNA XIST Inhibits Cell Proliferation, Migration, Invasion and EMT by Regulating miR-212-3p/CBLL1 axis in Non-small Cell Lung Cancer Cells. Eur. Rev. Med. Pharmacol. Sci. 23, 8391–8402. 10.26355/eurrev_201910_19150 31646569

[B73] Rieken M. Shariat S. F. Kluth L. A. Fajkovic H. Rink M. Karakiewicz P. I. (2015). Association of Cigarette Smoking and Smoking Cessation with Biochemical Recurrence of Prostate Cancer in Patients Treated with Radical Prostatectomy. Eur. Urol. 68, 949–956. 10.1016/j.eururo.2015.05.038 26050111

[B74] Robays L. J. Lanckacker E. A. Moerloose K. B. Maes T. Bracke K. R. Brusselle G. G. (2009). Concomitant Inhalation of Cigarette Smoke and Aerosolized Protein Activates Airway Dendritic Cells and Induces Allergic Airway Inflammation in a TLR-independent Way. J. Immunol. 183, 2758–2766. 10.4049/jimmunol.0802204 19635922

[B75] Robinson M. D. McCarthy D. J. Smyth G. K. (2010). edgeR: a Bioconductor Package for Differential Expression Analysis of Digital Gene Expression Data. Bioinformatics 26, 139–140. 10.1093/bioinformatics/btp616 19910308 PMC2796818

[B76] Salk J. J. Fox E. J. Loeb L. A. (2010). Mutational Heterogeneity in Human Cancers: Origin and Consequences. Annu. Rev. Pathol. Mech. Dis. 5, 51–75. 10.1146/annurev-pathol-121808-102113 PMC3375045 19743960

[B77] Sardari Nia P. Weyler J. Colpaert C. Vermeulen P. Marck E. V. Schil P. V. (2005). Prognostic Value of Smoking Status in Operated Non-small Cell Lung Cancer. Lung Cancer. 47, 351–359. 10.1016/j.lungcan.2004.08.011 15713518

[B78] Schaal C. M. Bora-Singhal N. Kumar D. M. Chellappan S. P. (2018). Regulation of Sox2 and Stemness by Nicotine and Electronic-Cigarettes in Non-small Cell Lung Cancer. Mol. Cancer. 17, 149. 10.1186/s12943-018-0901-2 30322398 PMC6190543

[B79] Schröder M. S. Culhane A. C. Quackenbush J. Haibe-Kains B. (2011). Survcomp: an R/Bioconductor Package for Performance Assessment and Comparison of Survival Models. Bioinformatics 27, 3206–3208. 10.1093/bioinformatics/btr511 21903630 PMC3208391

[B80] Shang Y. Li X. Liu W. Shi X. Yuan S. Huo R. (2020). Comprehensive Genomic Profile of Chinese Lung Cancer Patients and Mutation Characteristics of Individuals Resistant to Icotinib/gefitinib. Sci. Rep. 10, 20243. 10.1038/s41598-020-76791-y 33219256 PMC7679461

[B81] Shen R. Li P. Li B. Zhang B. Feng L. Cheng S. (2019). Identification of Distinct Immune Subtypes in Colorectal Cancer Based on the Stromal Compartment. Front. Oncol. 9, 1497. 10.3389/fonc.2019.01497 31998649 PMC6965328

[B82] Shen T. Le W. Yee A. Hwang P. H. Upadhyay D. Upadhyay D. (2010). Nicotine Induces Resistance to Chemotherapy in Nasal Epithelial Cancer. Am. J. Rhinol allergy 24, e73–e77. 10.2500/ajra.2010.24.3456 20338106

[B83] Shi W. Gerster K. Alajez N. M. Tsang J. Waldron L. Pintilie M. (2011). MicroRNA-301 Mediates Proliferation and Invasion in Human Breast Cancer. Cancer Res. 71, 2926–2937. 10.1158/0008-5472.CAN-10-3369 21393507

[B84] Shiels M. S. Katki H. A. Freedman N. D. Purdue M. P. Wentzensen N. Trabert B. (2014). Cigarette Smoking and Variations in Systemic Immune and Inflammation Markers. J. Natl. Cancer Inst. 106, 106. 10.1093/jnci/dju294 PMC4200029 25274579

[B85] Shinawi T. Hill V. K. Krex D. Schackert G. Gentle D. Morris M. R. (2013). DNA Methylation Profiles of Long- and Short-Term Glioblastoma Survivors. Epigenetics 8, 149–156. 10.4161/epi.23398 23291739 PMC3592900

[B86] Singh K. P. Maremanda K. P. Li D. Rahman I. (2020). Exosomal microRNAs Are Novel Circulating Biomarkers in Cigarette, Waterpipe Smokers, E-Cigarette Users and Dual Smokers. BMC Med. Genomics 13, 128. 10.1186/s12920-020-00748-3 32912198 PMC7488025

[B87] Soler P. Moreau A. Basset F. Hance A. J. (1989). Cigarette Smoking-Induced Changes in the Number and Differentiated State of Pulmonary Dendritic Cells/Langerhans Cells. Am. Rev. Respir. Dis. 139, 1112–1117. 10.1164/ajrccm/139.5.1112 2712439

[B88] Stang A. Knowlton R. Rekowski J. Gershman S. T. Galea S. (2021). Smoking Cessation Potential Among Newly Diagnosed Cancer Patients: a Population-Based Study of the Ten Most Common Cancers in Massachusetts, USA, 2008-2013. Ann. Epidemiol. 56, 55–60. 10.1016/j.annepidem.2020.11.004 33189878

[B89] Stánitz É. Juhász K. Gombos K. Gőcze K. Tóth C. Kiss I. (2015). Alteration of miRNA Expression Correlates with Lifestyle, Social and Environmental Determinants in Esophageal Carcinoma. Anticancer Res. 35, 1091–1097. 25667498

[B90] Sun J. Xu Z. Lv H. Wang Y. Wang L. Ni Y. (2018). eIF5A2 Regulates the Resistance of Gastric Cancer Cells to Cisplatin via Induction of EMT. Am. J. Transl Res. 10, 4269–4279. 30662669 PMC6325524

[B91] Sun X. Song J. Li E. Geng H. Li Y. Yu D. (2020). Cigarette Smoke Supports Stemness and Epithelial-Mesenchymal Transition in Bladder Cancer Stem Cells through SHH Signaling. Int. J. Clin. Exp. Pathol. 13, 1333–1348. 32661469 PMC7344017

[B92] Taioli E. (2008). Gene-environment Interaction in Tobacco-Related Cancers. Carcinogenesis 29, 1467–1474. 10.1093/carcin/bgn062 18550573 PMC2733188

[B93] Takahashi H. Ogata H. Nishigaki R. Broide D. H. Karin M. (2010). Tobacco Smoke Promotes Lung Tumorigenesis by Triggering IKKβ- and JNK1-dependent Inflammation. Cancer Cell. 17, 89–97. 10.1016/j.ccr.2009.12.008 20129250 PMC2818776

[B94] Thorsson V. Gibbs D. L. Brown S. D. Wolf D. Bortone D. S. Ou Yang T. H. (2018). The Immune Landscape of Cancer. Immunity 48, 812–e14. 10.1016/j.immuni.2018.03.023 29628290 PMC5982584

[B95] Tsao A. S. Liu D. Lee J. J. Spitz M. Hong W. K. (2006). Smoking Affects Treatment Outcome in Patients with Advanced Nonsmall Cell Lung Cancer. Cancer. 106, 2428–2436. 10.1002/cncr.21884 16634096

[B96] Vähäkangas K. H. Bennett W. P. Castrén K. Welsh J. A. Khan M. A. Blömeke B. (2001). p53 and K-Ras Mutations in Lung Cancers from Former and Never-Smoking Women. Cancer Res. 61, 4350–4356. 11389059

[B97] Villard P.-H. Hcrber R. Sérée E. M. Attolini L. Magdalou J. Lacarelle B. (1998). Effect of Cigarette Smoke on UDP-Glucuronosyltransferase Activity and Cytochrome P450 Content in Liver, Lung and Kidney Microsomes in Mice. Pharmacol. Toxicol. 82, 74–79. 10.1111/j.1600-0773.1998.tb01401.x 9498235

[B98] Vineis P. Esteve J. Hartge P. Hoover R. Silverman D. T. Terracini B. (1988). Effects of Timing and Type of Tobacco in Cigarette-Induced Bladder Cancer. Cancer Res. 48, 3849–3852. 3378220

[B99] Vladimirov B. S. Schiodt M. (2009). The Effect of Quitting Smoking on the Risk of Unfavorable Events after Surgical Treatment of Oral Potentially Malignant Lesions. Int. J. Oral Maxillofac. Surg. 38, 1188–1193. 10.1016/j.ijom.2009.06.026 19640683

[B100] Waaijer C. J. F. de Andrea C. E. Hamilton A. van Oosterwijk J. G. Stringer S. E. Bovée J. V. M. G. (2012). Cartilage Tumour Progression Is Characterized by an Increased Expression of Heparan Sulphate 6O-Sulphation-Modifying Enzymes. Virchows Arch. 461, 475–481. 10.1007/s00428-012-1300-5 22903264

[B101] Wang L. Li Q. Ye Z. Qiao B. (2019). ZBTB7/miR-137 Autoregulatory Circuit Promotes the Progression of Renal Carcinoma. Oncol. Res. 27, 1007–1014. 10.3727/096504018X15231148037228 29673422 PMC7848413

[B102] Wang W. Liu M. Guan Y. Wu Q. (2016). Hypoxia-Responsive Mir-301a and Mir-301b Promote Radioresistance of Prostate Cancer Cells via Downregulating NDRG2. Med. Sci. Monit. 22, 2126–2132. 10.12659/msm.896832 27327120 PMC4920099

[B103] Willey J. C. Coy E. L. Frampton M. W. Torres A. Apostolakos M. J. Hoehn G. (1997). Quantitative RT-PCR Measurement of Cytochromes P450 1A1, 1B1, and 2B7, Microsomal Epoxide Hydrolase, and NADPH Oxidoreductase Expression in Lung Cells of Smokers and Nonsmokers. Am. J. Respir. Cell Mol Biol. 17, 114–124. 10.1165/ajrcmb.17.1.2783 9224217

[B104] Wu X. Chen H. Zhang G. Wu J. Zhu W. Gu Y. (2019). MiR-212-3p Inhibits Cell Proliferation and Promotes Apoptosis by Targeting Nuclear Factor IA in Bladder Cancer. J. Biosci. 44, 44. 10.1007/s12038-019-9903-5 31502558

[B105] Wu Z.-H. Cai F. Zhong Y. (2020). Comprehensive Analysis of the Expression and Prognosis for GBPs in Head and Neck Squamous Cell Carcinoma. Sci. Rep. 10, 6085. 10.1038/s41598-020-63246-7 32269280 PMC7142114

[B106] Yan T. Ye X. X. (2019). MicroRNA-328-3p Inhibits the Tumorigenesis of Bladder Cancer through Targeting ITGA5 and Inactivating PI3K/AKT Pathway. Eur. Rev. Med. Pharmacol. Sci. 23, 5139–5148. 10.26355/eurrev_201906_18178 31298367

[B107] Yoo H.-I. Kim B.-K. Yoon S. K. (2016). MicroRNA-330-5p Negatively Regulates ITGA5 Expression in Human Colorectal Cancer. Oncol. Rep. 36, 3023–3029. 10.3892/or.2016.5092 27633518

[B108] Yoshihara K. Shahmoradgoli M. Martínez E. Vegesna R. Kim H. Torres-Garcia W. (2013). Inferring Tumour Purity and Stromal and Immune Cell Admixture from Expression Data. Nat. Commun. 4, 2612. 10.1038/ncomms3612 24113773 PMC3826632

[B109] Yu X. J. Chen G. Yang J. Yu G. C. Zhu P. F. Jiang Z. K. (2019). Smoking Alters the Evolutionary Trajectory of Non-small C-ell L-ung C-ancer. Exp. Ther. Med. 18, 3315–3324. 10.3892/etm.2019.7958 31602204 PMC6777332

[B110] Yuge K. Kikuchi E. Hagiwara M. Yasumizu Y. Tanaka N. Kosaka T. (2015). Nicotine Induces Tumor Growth and Chemoresistance through Activation of the PI3K/Akt/mTOR Pathway in Bladder Cancer. Mol. Cancer Ther. 14, 2112–2120. 10.1158/1535-7163.MCT-15-0140 26184482

[B111] Zeng Z. Lei S. He Z. Chen T. Jiang J. (2021). YEATS2 Is a Target of HIF1α and Promotes Pancreatic Cancer Cell Proliferation and Migration. J. Cell Physiol. 236, 2087–2098. 10.1002/jcp.29995 32749678

[B112] Zeybel M. Vatansever S. Hardy T. Sarı A. A. Cakalağaoğlu F. Avcı A. (2016). DNA Methylation Profiling Identifies Novel Markers of Progression in Hepatitis B-Related Chronic Liver Disease. Clin. Epigenet. 8, 48. 10.1186/s13148-016-0218-1 PMC4857425 27152124

[B113] Zhang T. Wang Z. Liu Y. Huo Y. Liu H. Xu C. (2020). Plastin 1 Drives Metastasis of Colorectal Cancer through the IQGAP1/Rac1/ERK Pathway. Cancer Sci. 111, 2861–2871. 10.1111/cas.14438 32350953 PMC7419044

[B114] Zhang Y. Ma A. Wang L. Zhao B. (2015). Nornicotine and Nicotine Induced Neovascularization via Increased VEGF/PEDF Ratio. Ophthalmic Res. 55, 1–9. 10.1159/000440847 26536586

[B115] Zhou W. Heist R. S. Liu G. Park S. Neuberg D. S. Asomaning K. (2006). Smoking Cessation before Diagnosis and Survival in Early Stage Non-small Cell Lung Cancer Patients. Lung Cancer. 53, 375–380. 10.1016/j.lungcan.2006.05.017 16814423

