# Supplementary figures and images for: Multi-Omics Analysis of the Effects of Smoking on Human Tumors
Source: Front Mol Biosci. 2021 Nov 2;8:704910. doi: 10.3389/fmolb.2021.704910 (PMC8592943; doi:10.3389/fmolb.2021.704910)

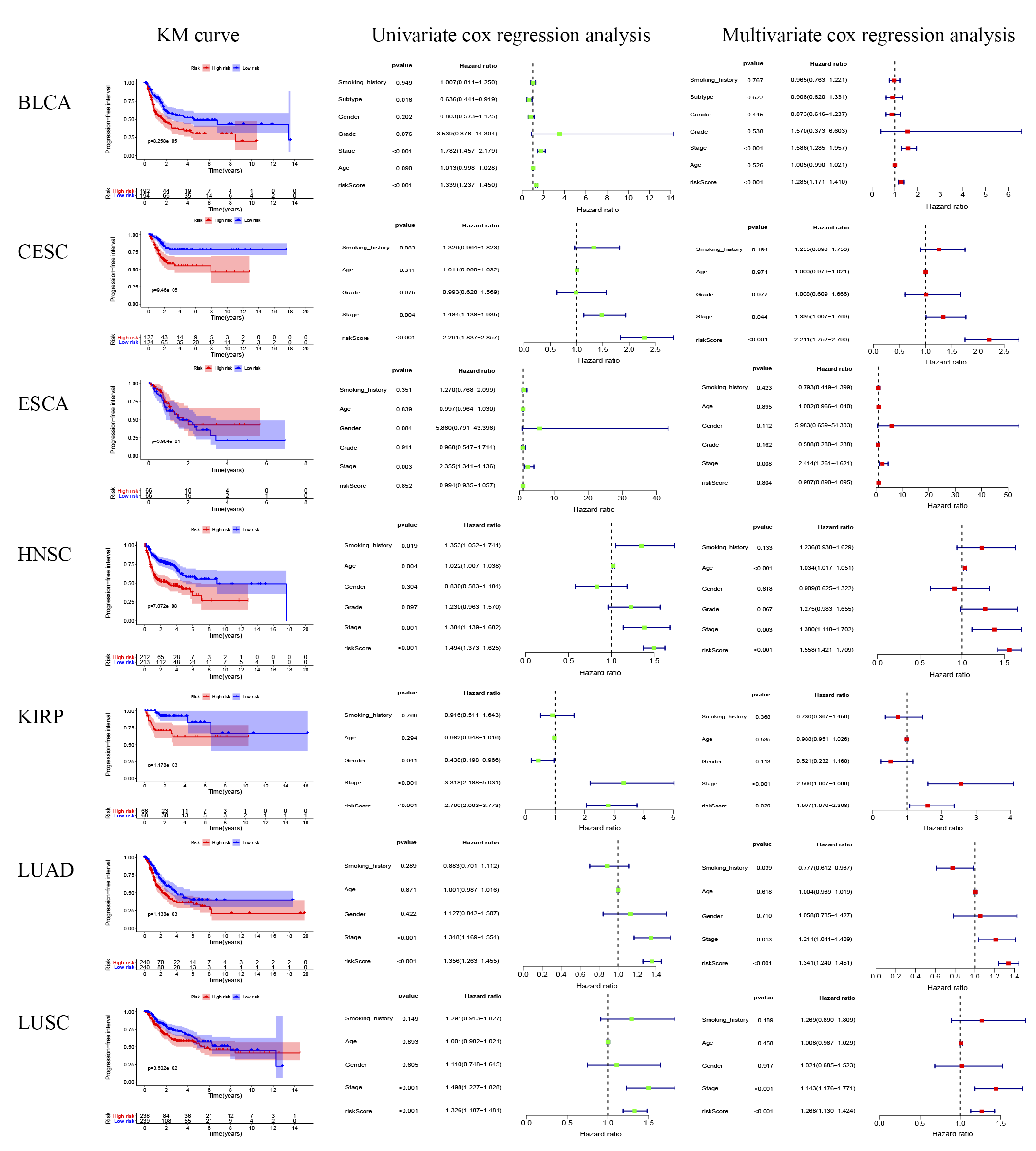

Supplement: Supplementary file 1 [file Image6.TIF]

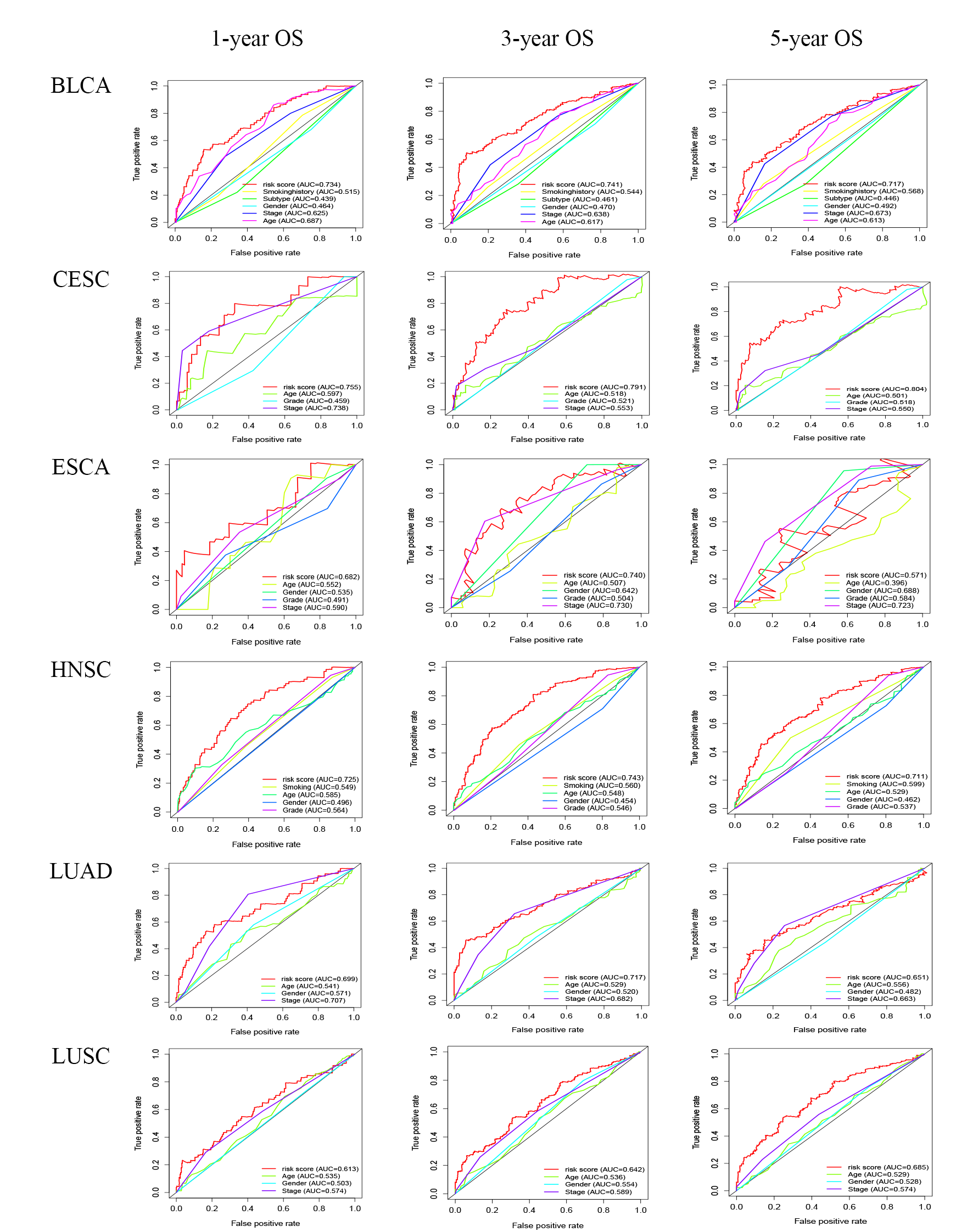

Supplement: Supplementary file 2 [file Image3.TIF]

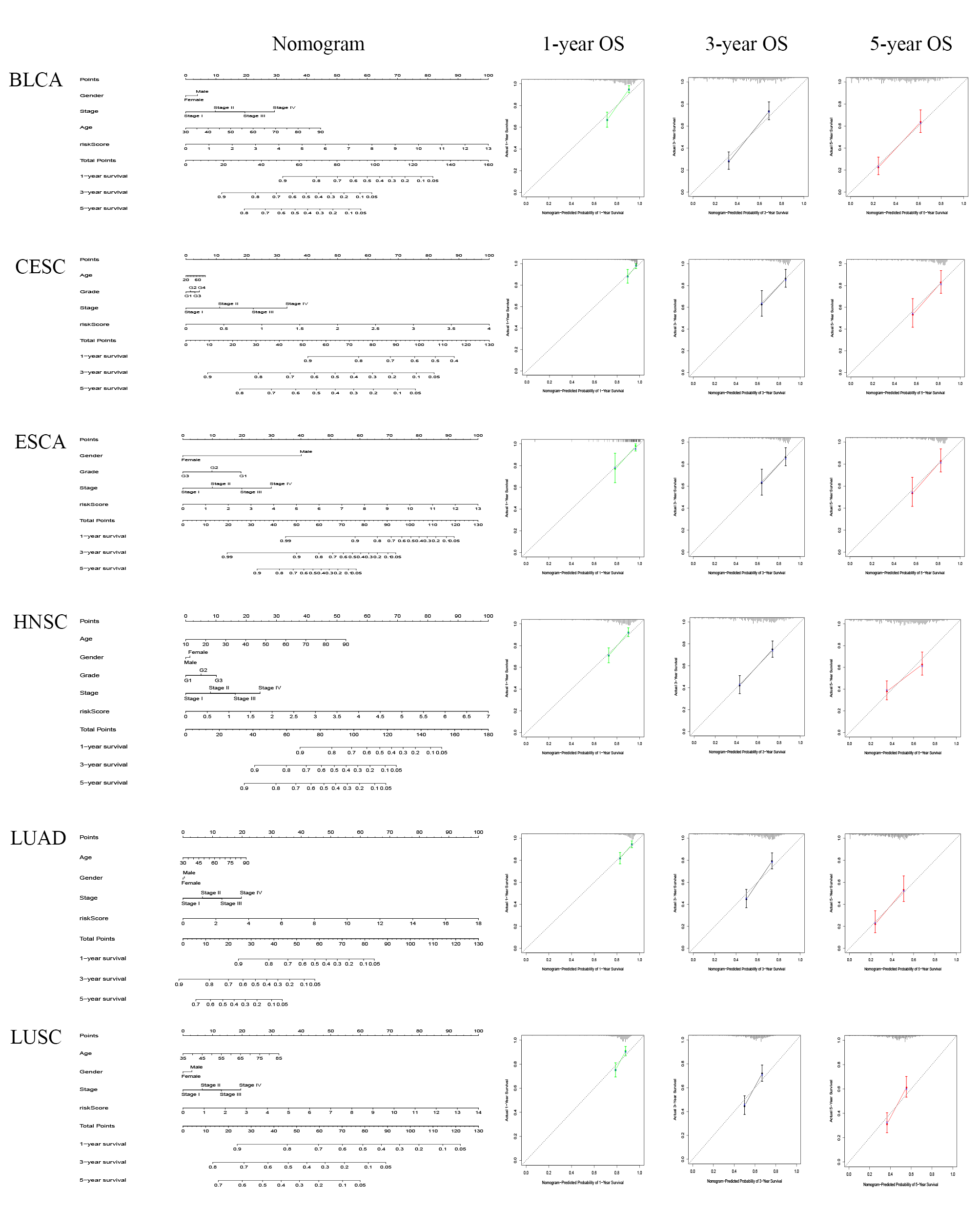

Supplement: Supplementary file 3 [file Image4.TIF]

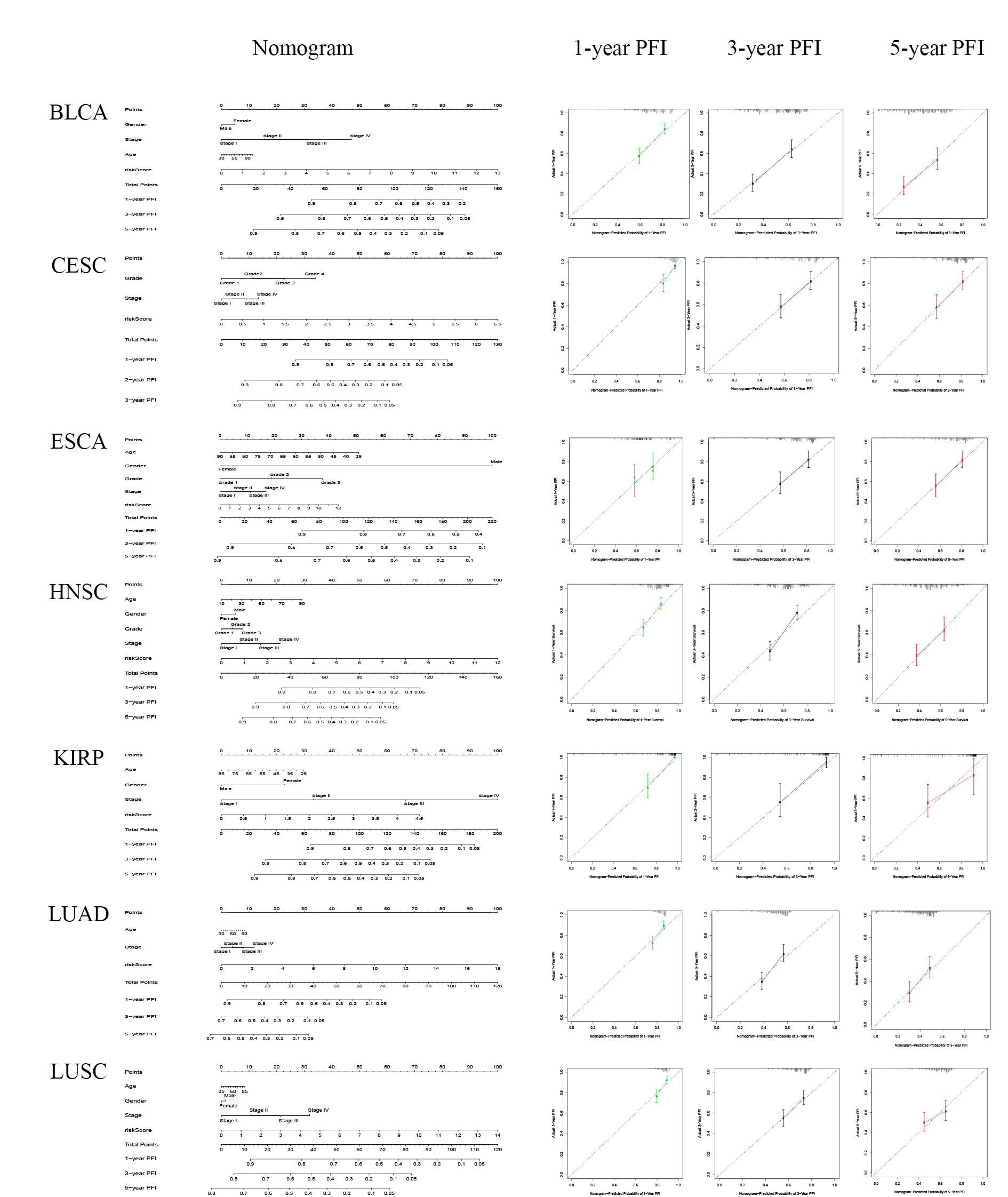

Supplement: Supplementary file 4 [file Image9.TIF]

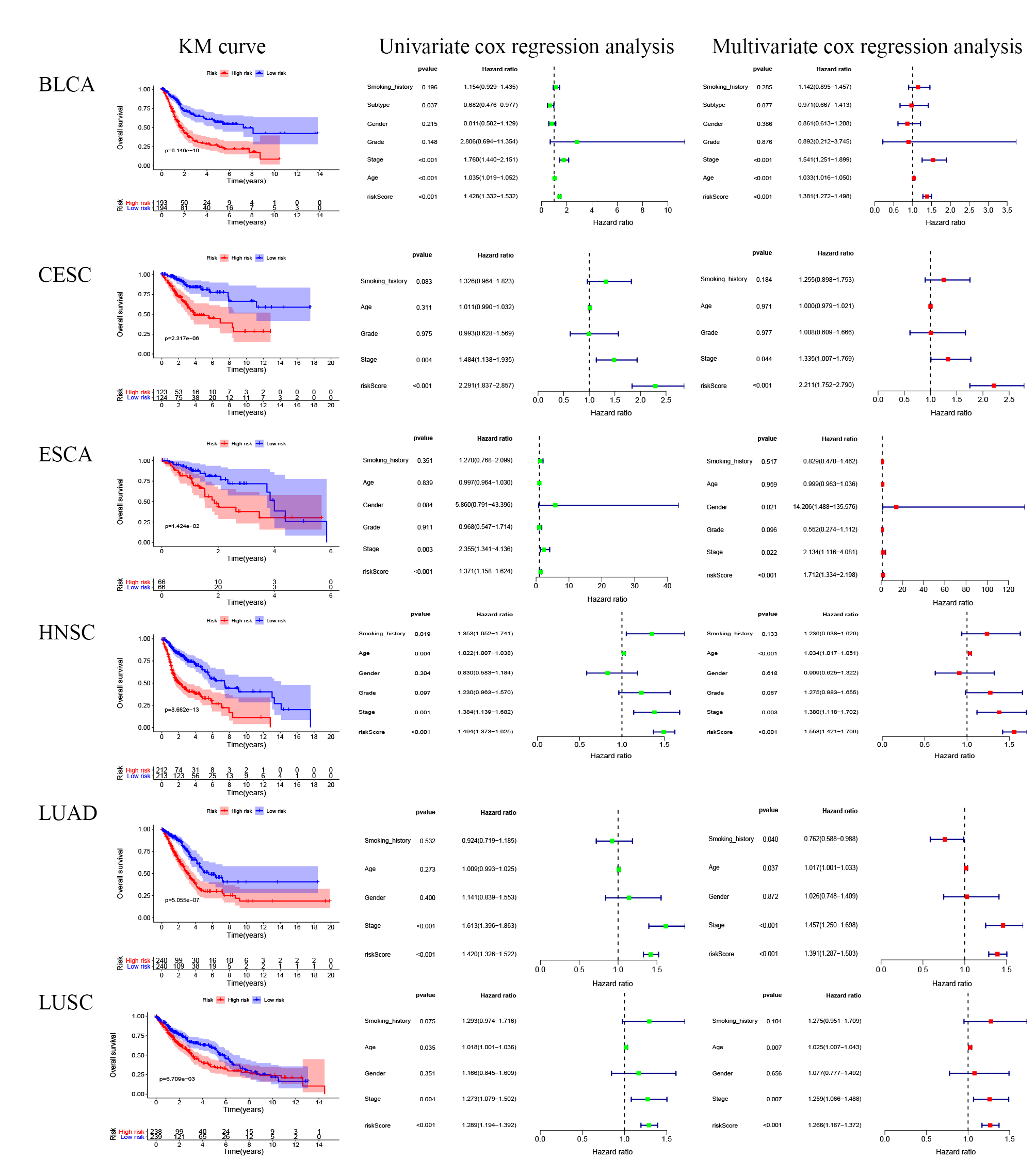

Supplement: Supplementary file 5 [file Image2.TIF]

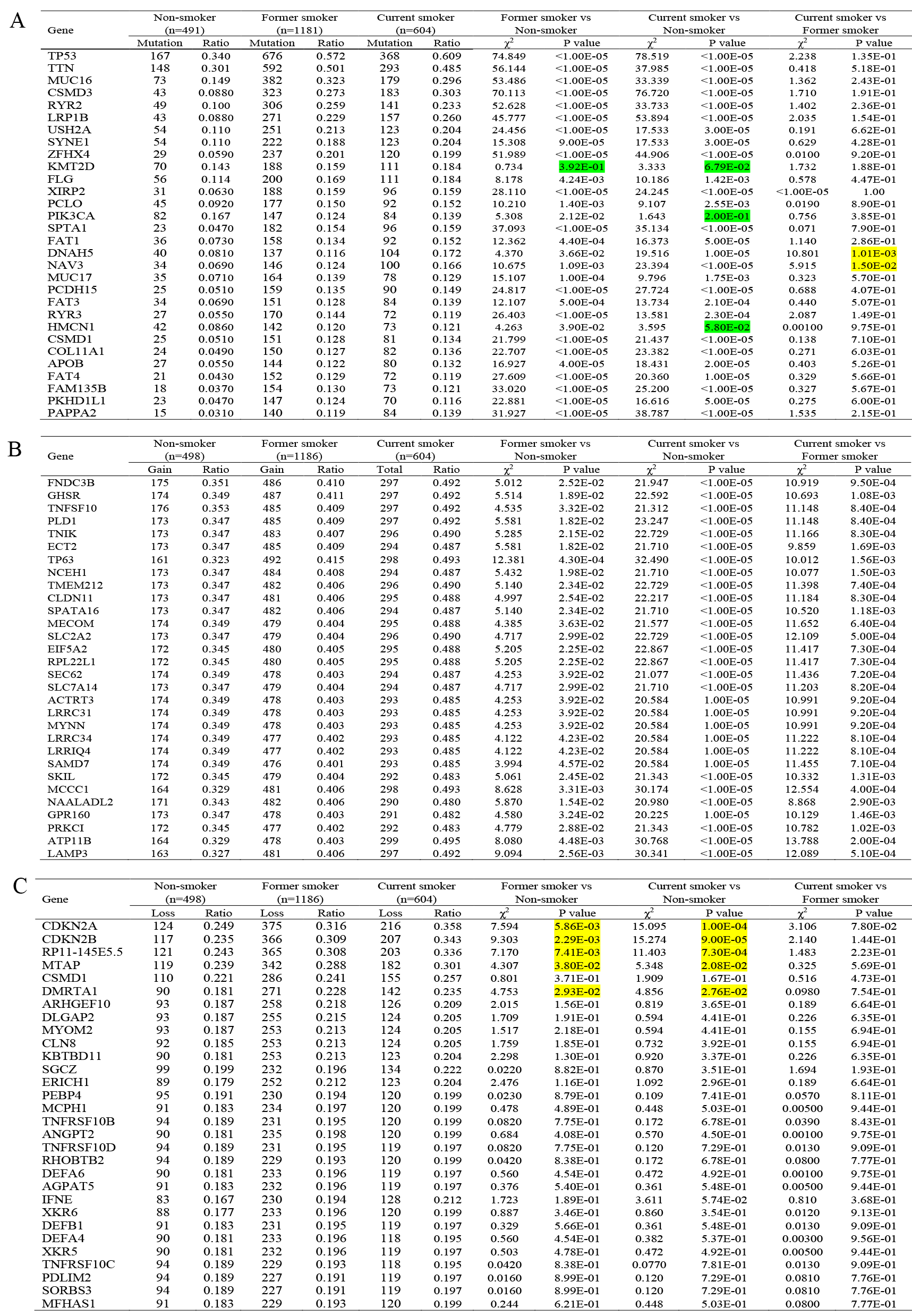

Supplement: Supplementary file 6 [file Image1.tif]

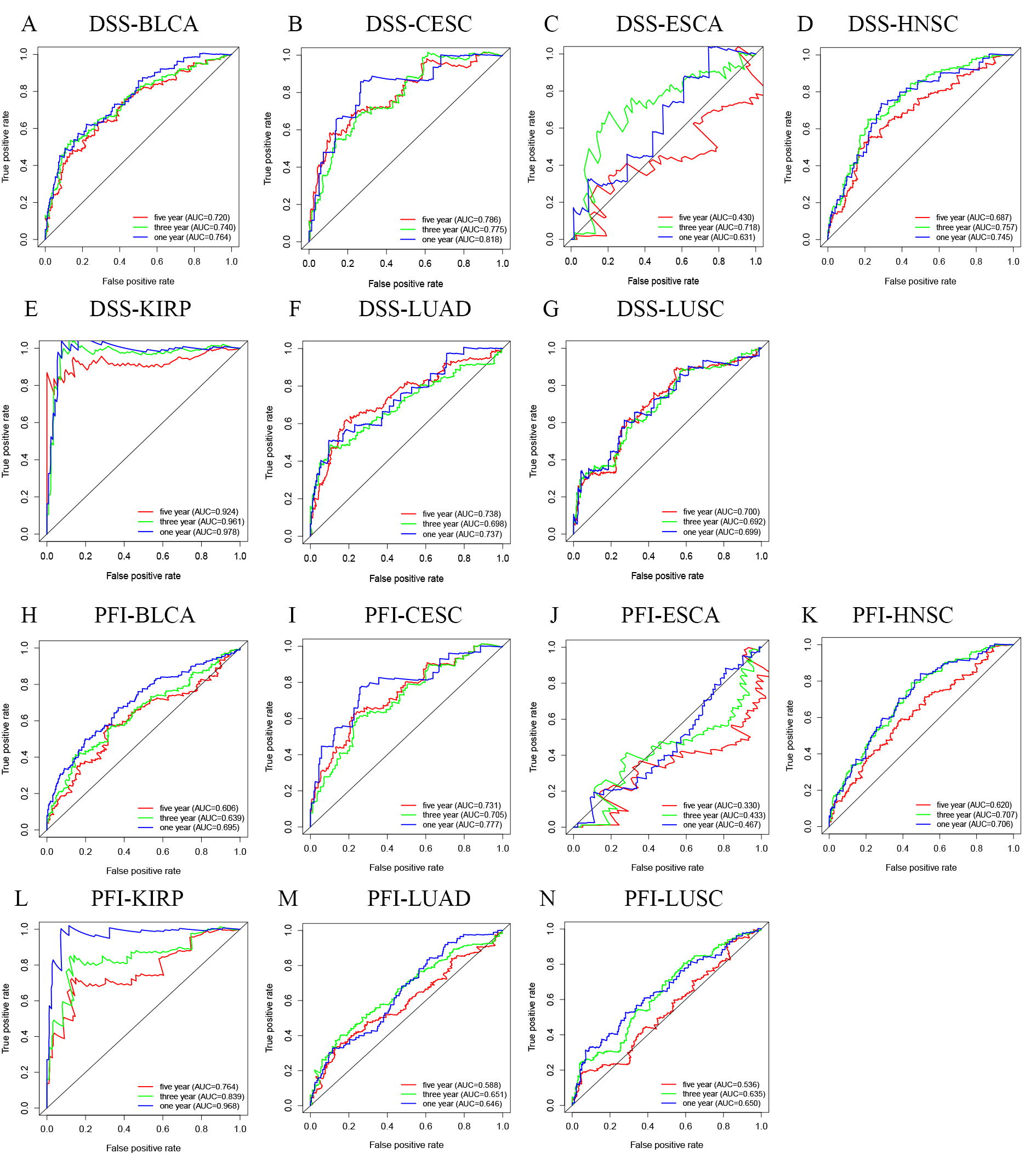

Supplement: Supplementary file 7 [file Image7.TIF]

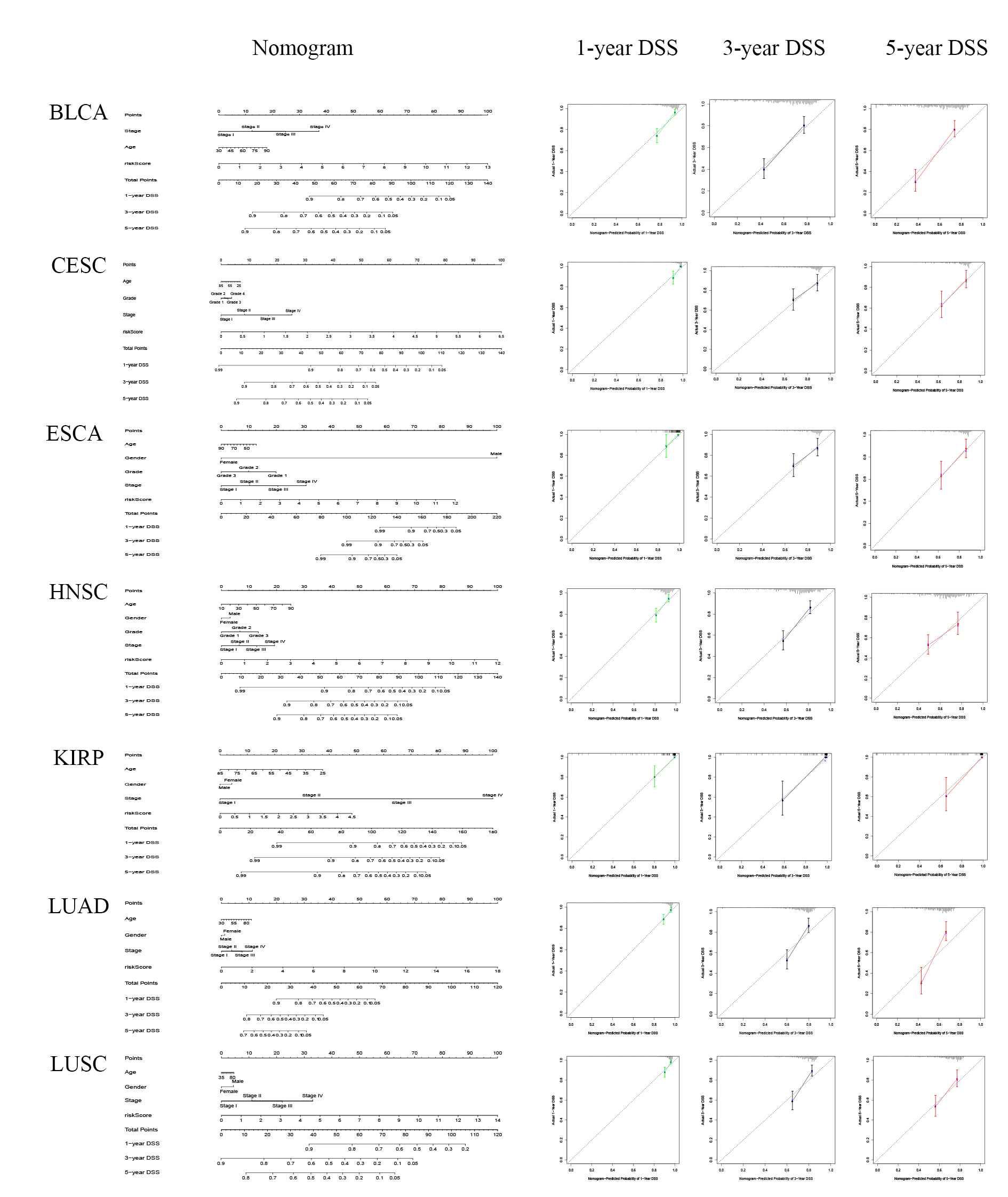

Supplement: Supplementary file 8 [file Image8.TIF]

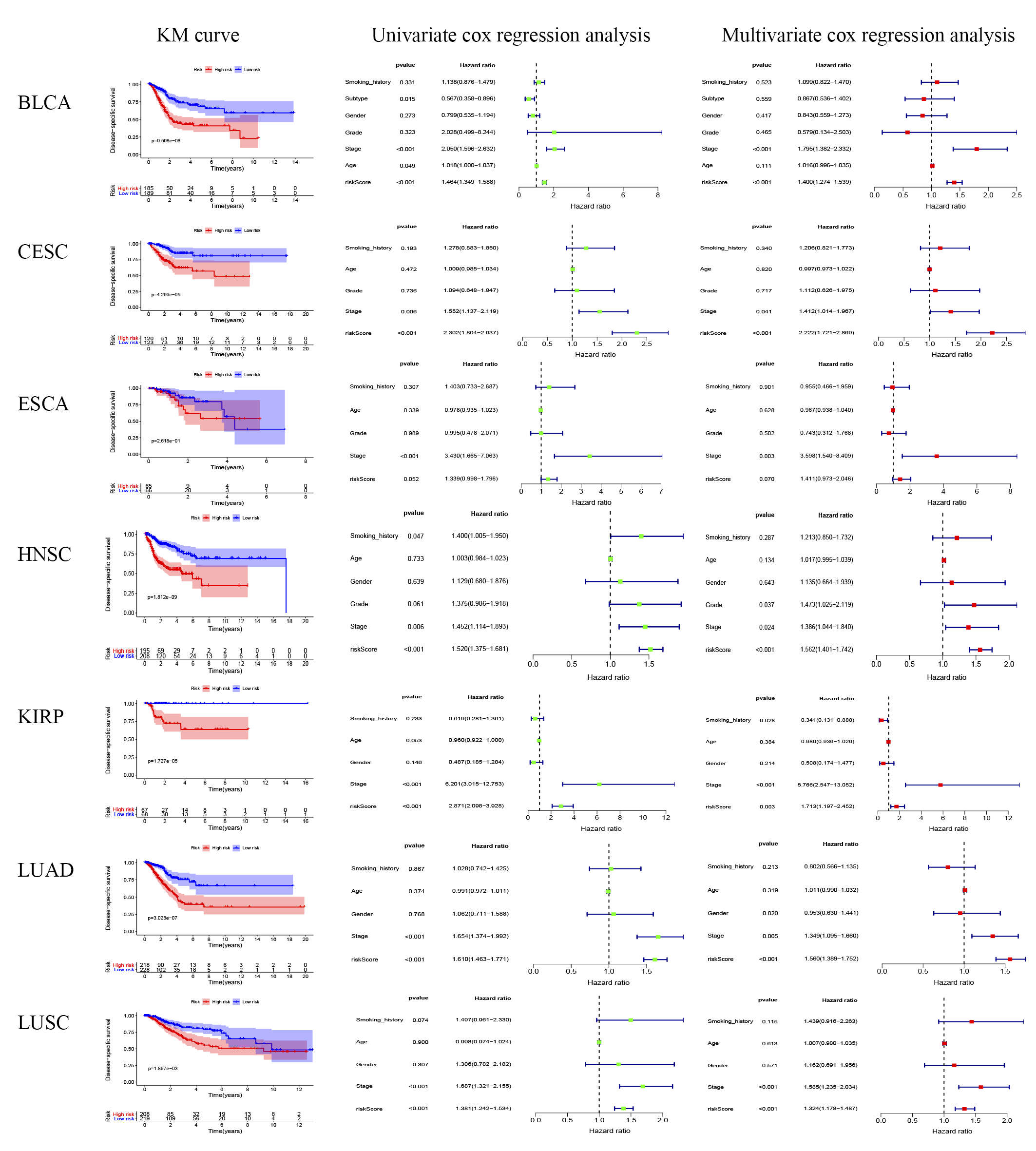

Supplement: Supplementary file 9 [file Image5.TIF]
